# Runaway GC Evolution in Gerbil Genomes

**DOI:** 10.1093/molbev/msaa072

**Published:** 2020-04-24

**Authors:** Rodrigo Pracana, Adam D Hargreaves, John F Mulley, Peter W H Holland

**Affiliations:** m1 Department of Zoology, University of Oxford, Oxford, United Kingdom; m2 School of Natural Sciences, Bangor University, Bangor, Gwynedd, United Kingdom

**Keywords:** GC-content, GC-biased gene conversion, gBGC, recombination, genome evolution, biased substitution, fixation bias

## Abstract

Recombination increases the local GC-content in genomic regions through GC-biased gene conversion (gBGC). The recent discovery of a large genomic region with extreme GC-content in the fat sand rat *Psammomys obesus* provides a model to study the effects of gBGC on chromosome evolution. Here, we compare the GC-content and GC-to-AT substitution patterns across protein-coding genes of four gerbil species and two murine rodents (mouse and rat). We find that the known high-GC region is present in all the gerbils, and is characterized by high substitution rates for all mutational categories (AT-to-GC, GC-to-AT, and GC-conservative) both at synonymous and nonsynonymous sites. A higher AT-to-GC than GC-to-AT rate is consistent with the high GC-content. Additionally, we find more than 300 genes outside the known region with outlying values of AT-to-GC synonymous substitution rates in gerbils. Of these, over 30% are organized into at least 17 large clusters observable at the megabase-scale. The unusual GC-skewed substitution pattern suggests the evolution of genomic regions with very high recombination rates in the gerbil lineage, which can lead to a runaway increase in GC-content. Our results imply that rapid evolution of GC-content is possible in mammals, with gerbil species providing a powerful model to study the mechanisms of gBGC.

## Introduction

The base composition of DNA shows considerable variation within genomes and between species. For example, genes and genomes can differ strikingly in GC-content, defined as the proportion of G and C nucleotides in a sequence of DNA. An important topic in evolutionary genetics is understanding the processes that lead to changes in GC-content, particularly those with direct effects on functional genetic elements (Marais et al. 2001; Galtier and Duret 2007) or on chromatin structure (Vinogradov 2003).

Broadly speaking, the GC-content of a species is thought to result from the balance between mutation, which is generally AT-biased (Lynch 2007; Long et al. 2018), and a type of homologous recombination known as gene conversion, which is generally GC-biased (Lamb 1984; Galtier et al. 2001; Galtier and Duret 2007; Duret and Galtier 2009; Odenthal-Hesse et al. 2014). Gene conversion takes place during meiosis, through the repair of double-strand breaks. Meiotic double-strand breaks occur on a chromosome at sites where it meets its homologous chromosome, resulting either in crossovers or in noncrossovers (Arnheim et al. 2007; Cole et al. 2010). Their repair involves the use of the homologous chromosome as a template for resynthesis and replacement of a short stretch of DNA on the broken chromosome. In the cases where the double-strand break occurs near a heterozygous site, the broken chromosome can thus be “converted,” that is, it can receive the allele of the homologous chromosome and lose the original allelic variant. Importantly, at GC:AT heterozygous sites the gene conversion of AT alleles to GC alleles occurs more often than the opposite conversion of GC alleles to AT alleles (Lamb 1984; Galtier et al. 2001; Galtier and Duret 2007; Duret and Galtier 2009; Smeds et al. 2016). Indeed, AT (“weak”) to GC (“strong”) conversions have been shown to occur in 68% of observable gene conversions of GC:AT heterozygous sites in humans and mice (Odenthal-Hesse et al. 2014; Williams et al. 2015; Halldorsson et al. 2016; Li et al. 2019). This nonrandom transmission of GC alleles between homologous chromosomes is known as GC-biased gene conversion (gBGC).

In genomic regions with high recombination rates—that is, those with a high density of double-strand break hotspots—gBGC causes the frequency of G and C alleles to increase over time (Eyre-Walker 1999; Webster and Smith 2004; Spencer et al. 2006; Katzman et al. 2011; Auton et al. 2012). As a consequence, a correlation is expected between the rate of recombination and the rate of weak-to-strong (AT-to-GC) substitutions between species (Duret and Arndt 2008). Studying the genomes of recently diverged species, such as human and chimpanzee, shows that there is a correlation between the weak-to-strong (AT-to-GC) substitution rate and both the current and the reconstructed ancestral rates of recombination (Dreszer et al. 2007; Capra et al. 2013; Munch et al. 2014). In birds, where karyotypes and recombination rates are exceptionally conserved (Singhal et al. 2015), the correlation has been detected even at longer phylogenetic distances (Mugal et al. 2013; Botero-Castro et al. 2017; Corcoran et al. 2017; Bolívar et al. 2019; Rousselle et al. 2019). Additionally, GC-content has been shown to be correlated with indirect proxies of recombination rate, for instance chromosome length, with species with smaller chromosomes having on average a higher recombination rate and therefore, higher GC-content (Romiguier et al. 2010; Nabholz et al. 2011; Pessia et al. 2012; Figuet et al. 2015). An implication of this gBGC hypothesis is that lineage-specific changes to GC-content are likely to have been caused by lineage-specific changes to the recombination landscape (Romiguier et al. 2010). Thus, lineages that are affected by extreme levels of GC-biased evolution are likely to be useful models in the study of the evolution of recombination.

One such species is a gerbil, *Psammomys obesus*, the fat sand rat. A recent study has shown that the genome of this species has an unusual region with an extremely high GC-content (Hargreaves et al. 2017). This region contains at least 88 genes, including several that are highly conserved across mammal species, such as *Brca2*, *Cdk8*, *Insr*, and the ParaHox cluster (*Gsx1*, *Pdx1*, *Cdx2*), and is syntenic to the subtelomeric region of chromosome 12 in rat. The region had previously eluded study because of the difficulty of sequencing DNA sequences with very high GC-content when the rest of a sample is not GC-rich (Chen et al. 2013; Botero-Castro et al. 2017). Using transcriptome sequencing, Hargreaves et al. (2017) assembled 52 out of 88 known genes in the region and showed that a subset of at least 30 had a GC-content greater than their homologues in the mouse and rat, to which gerbils are closely related. By focusing on the *Pdx1* gene, they also showed that the region is affected by extreme nonsynonymous evolution: The 60 amino-acid homeodomain of PDX1 is 100% conserved across all previously studied mammals, yet in *P. obesus* it differs by 15 amino acids. Because of the considerable level of conservation of this protein across vertebrates, it is likely that most of these changes were deleterious when they originated in *P. obesus* (Hargreaves et al. 2017; Dai and Holland 2019). The implication is that the region has evolved to this state not because of selection for high GC-content, but through a process that increases GC-content regardless of deleterious effects of the G or C alleles (Berglund et al. 2009; Galtier et al. 2009; Ratnakumar et al. 2010; Kostka et al. 2012; Dai and Holland 2019). The suggestion is that an expansive genomic region has been affected by anomalously stable and intense gBGC, causing extreme sequence divergence in some genes in a short period of time (the most recent common ancestor of mice and gerbils lived between 20.6 and 22.5 Ma; Steppan et al. 2004). We reasoned that this rapid evolution of localized GC-content affords a powerful opportunity to explore the molecular drivers underpinning GC evolution in mammalian genomes.

In this study, we test whether gBGC was responsible for the increase in GC-content by measuring the substitution rates for weak-to-strong (AT-to-GC), strong-to-weak (GC-to-AT), and GC-conservative mutations in the fat sand rat *P. obesus*, as well as in three additional species from the gerbil clade (subfamily Gerbillinae). We hypothesized that the region evolved on the gerbil stem lineage, hence we measured the substitution rates from the point of Gerbillinae–Murinae divergence, and compared rates with those measured for two murine species, the mouse *Mus musculus* and the rat *Rattus norvegicus.* We tested whether the high-GC region is affected by a high rate of weak-to-strong (AT-to-GC) substitutions, but not of other types of substitution, as expected if the high GC in the region results from gBGC alone. Finally, we explore whether other parts of the gerbil genome are affected by similar GC-skewed evolution, as predicted if there was a global change in recombination.

## Results

### All Mutational Categories Have an Increased Substitution Rate in the Known High-GC Region

**Fig. 1.**
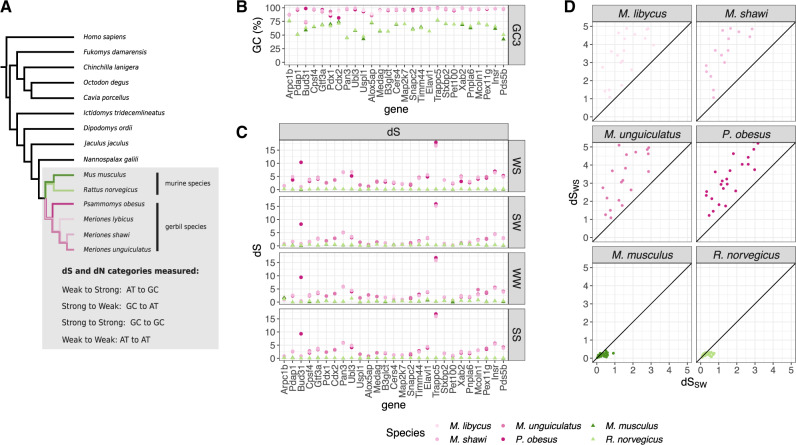
GC-skewed synonymous evolution in protein-coding genes located in the known high-GC region. (*A*) Topology of the phylogenetic relationships between the species analyzed in this study; (*B*) GC-content in the third-codon position (GC3) per species per gene; (*C*) the rate of synonymous substitution (d*S*) per mutational category: weak-to-strong (WS), strong-to-weak (SW), weak-to-weak (WW), and strong-to-strong (SS); (*D*) comparison between weak-to-strong d*S* (d*S*_WS_) and strong-to-weak d*S* (d*S*_SW_).

Our first aim was to test whether a previously described high-GC region is found in the genomes of gerbil species other than the sand rat *P. obesus*. We sequenced and assembled transcriptomes from three gerbil species (*Meriones unguiculatus*, *M. libycus*, and *M. shawi*, fig. 1*A*). From each species, we identified orthologues of the protein-coding genes located in the known high-GC region of the sand rat, and compared them with orthologous genes in *P. obesus* and in two murine species, *M. musculus* and *R. norvegicus*. This gave a subset of 27 genes with representative sequences in at least one of the four gerbil species (20 genes had a sequence in all four gerbil species). For each gene and species, we measured GC in the third-codon position (GC3). Consistent with previous observations, the genes in the region had an extremely high GC3 in *P. obesus*, ranging from 81% to 100%, compared with the 42–77% in *M. musculus* and 45–78% in *R. norvegicus* (fig. 1*B*). In the three other gerbil species, GC3 was also extremely high, ranging from 73% to 100% in *M. unguiculatus*, 74% to 100% in *M. libycus*, and 75% to 99% in *M. shawi* (fig. 1*B*). The gene sequences of the gerbil species represented in each of the 27 genes had, in all cases, a higher GC3 value than the sequences of the two murine species. This result supports the hypothesis that the region of high GC is not unique to the fat sand rat, but evolved before the divergence between the four gerbil species represented in our samples.

A hypothesis that could explain the high GC3 in the region is that the fixation rate of G or C alleles is higher than that of A and T alleles, through a process, such as gBGC. gBGC is expected to directly increase the rate of AT-to-GC substitutions (weak-to-strong) yet to have no direct effect on the rate of GC-to-AT (strong-to-weak) or GC-conservative (strong-to-strong and weak-to-weak) substitutions (Duret and Arndt 2008). We tested whether the genes in the high-GC region of gerbils have encountered an increase in weak-to-strong substitution rate by estimating the rate of synonymous substitution (d*S*) for different mutational categories: Weak-to-strong (d*S*_WS_), strong-to-weak (d*S*_SW_), strong-to-strong (d*S*_SS_), and weak-to-weak (d*S*_WW_). We measured these values from the node of the tree representing the murine–gerbil divergence to the tips representing each of the four species (fig. 1*A*), thus measuring the divergence between the two groups of species (20.6–22.5 My of divergence). The value of d*S*_WS_ was greater than 1 for all genes in all gerbil species, compared with a range of just 0.03–0.4 in *M. musculus* and 0.04–0.32 in *R. norvegicus*. Surprisingly, however, the other three mutational categories also showed a higher d*S* in the gerbil species than in the murine species (fig. 1*C*) in all but three genes (*Arpc1b*, *Pet100*, and *Cers4*). Indeed, 20 of the 27 genes had d*S* >1 for all mutational categories in all gerbil species. These d*S* values observed in the gerbil species—and not in the murine species—indicate that, for most genes, the substitution rates of each mutational category are extreme and high enough to have reached saturation. Importantly, d*S*_WS_ was higher than d*S*_SW_ for all genes in the gerbil species (fig. 1*D*), consistent with the overall increase in GC for the genes in the region.

**Fig. 2.**
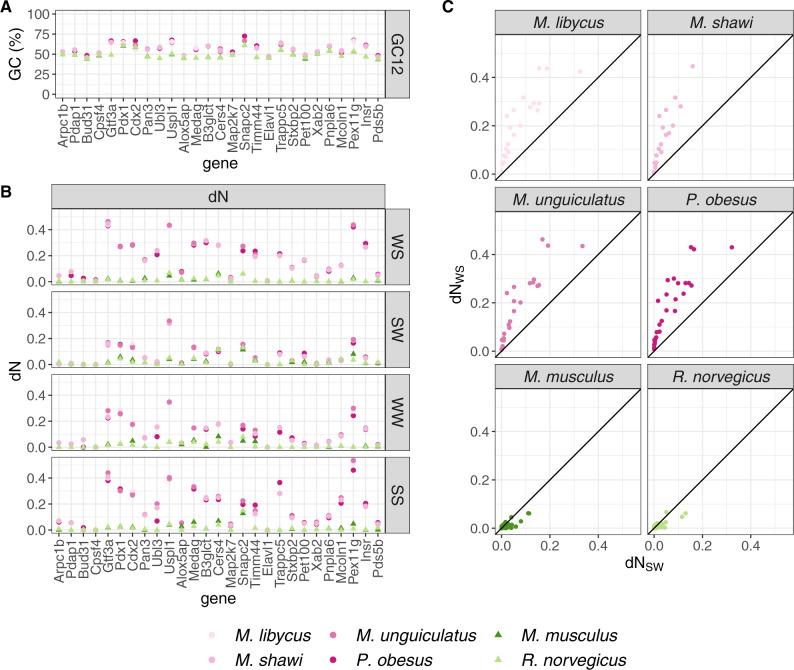
GC-skewed nonsynonymous evolution in protein-coding genes located in the known high-GC region. (*A*) GC-content in the first and second codon positions (GC12) per species per gene, (*B*) and nonsynonymous substitution rate (d*N*) per mutational category: weak-to-strong (WS), strong-to-weak (SW), weak-to-weak (WW), and strong-to-strong (SS), (*C*) comparison between weak-to-strong d*N* (d*N*_WS_) and strong-to-weak d*N* (d*N*_SW_).

These results are mirrored in the substitutions causing nonsynonymous changes. In 26 of the 27 genes, GC at the first and second codon position (GC12) had a higher value in the four gerbil species than in the two murine species (fig. 2*A*), despite both groups having overlapping ranges (45–72% in the gerbil species and 43–61% in the murine species). We measured the rate of nonsynonymous substitution (d*N*) for the four mutational categories: d*N*_WS_, d*N*_SW_, d*N*_SS_, and d*N*_WW_ (fig. 2*B*). The main difference between d*S* and d*N* is that d*N* was never greater than one for any of the categories. Otherwise, we detect the same patterns for d*N* and for d*S*. First, all 27 genes had greater d*N*_WS_ in the gerbil species than in the murine species (range 0.01–0.46 in the gerbil species and 0–0.07 in the murine species). Second, the three other categories also have elevated d*N* in the gerbil species relative to the mouse species in most genes (18 out of 27). Last, d*N*_WS_ was higher than d*N*_SW_ for all genes in the gerbil species (fig. 2*C*).

In summary, we found that the known region of high GC in gerbil species is characterized by an increase in d*S* and d*N* for all mutational categories, but with a higher rate of weak-to-strong substitutions than strong-to-weak substitutions.

### GC Skew Affects Other Genes in the Gerbil Genome

Our second aim was to test whether other genes in the genome of gerbils are affected by GC-skewed evolution, or whether the known GC-rich region is a unique peculiarity within gerbil genomes. Based on the results above, we sought to address this by asking three questions. 1) Are there genes with outlying d*S*_WS_ values in other parts of gerbil genomes? 2) If such genes exist, do they also have high d*S* values for the other three mutational categories? 3) Do these genes have a higher d*S*_WS_ than d*S*_SW_, thus being affected by GC-skewed evolution?

**Fig. 3.**
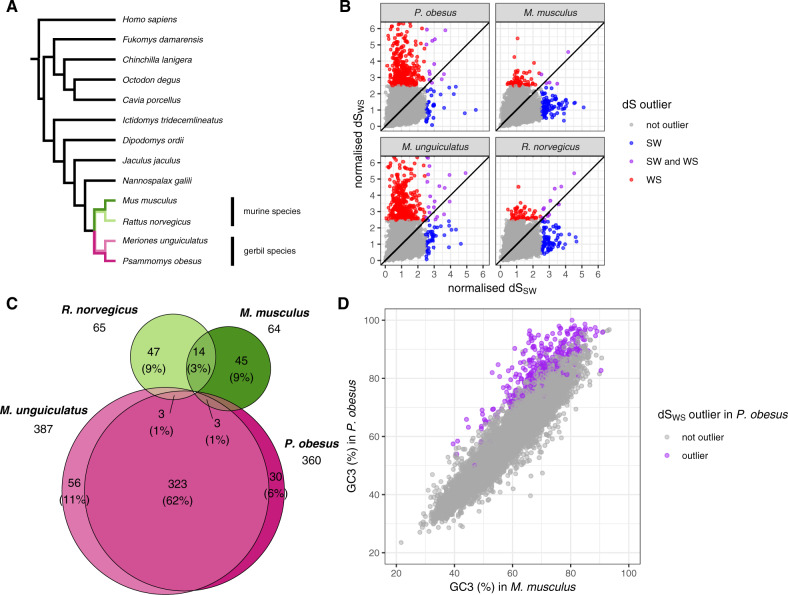
Outliers in the rate of synonymous substitution (d*S*) measured from the gerbil-murine split for 8,809 groups of orthologous genes outside the known high-GC region. (*A*) Topography of the tree of the species included in the alignments of each group of orthologous genes; our focal species include two gerbil species (in pink) and two murine species (in green); d*S* was measured in the colored branches, that is, from the point of gerbil-murine divergence to the tip of each of the four focal species. (*B*) Comparison between the rate of weak-to-strong synonymous substitution (d*S*_WS_) and the strong-to-weak synonymous substitution (d*S*_SW_) in the two gerbil and the two murine species, normalized by dividing each value by the average rate of the respective species and mutation category; colored points represent genes above the d*S* threshold chosen to define outliers (d*S* >2.5 times the average for the respective species and mutational category); only values under 6 are shown. (*C*) Euler diagram of the d*S*_WS_ outliers, where the area of each segment is approximately proportional to the number of overlapping outlier genes per species (only gene numbers >1 included), shown also as a percentage of the 524 genes that were outliers in at least one species. (*D*) Pairwise comparisons of the GC-content at the third-codon position (GC3) between the gerbil *Psammomys obesus* and the mouse *Mus musculus* for 8,797 groups of orthologous genes (out of 8,809) for which both species have a GC3 measurement.

To answer these questions, we identified 8,809 orthologous genes in a set of ten rodent species and in the outgroup *Homo sapiens*, excluding any genes from the known high-GC region. These groups of orthologous genes have a single copy in each of two gerbil species (*P. obesus* and *M. unguiculatus*) and two murine species (*M. musculus* and *R. norvegicus*). We measured the synonymous substitution rates of the different mutational categories (d*S*_WS_, d*S*_SW_, d*S*_WW_, and d*S*_SS_) from the node of the tree representing the murine–gerbil divergence to the tips representing each of the four species (fig. 3*A*). To control for different average evolutionary rates in each of the lineages, we divided each rate measurement by the average rate for the category and the species. For each of the mutational categories in each species, we considered genes with d*S* greater than 2.5 times the respective average as outliers, a threshold chosen to capture the tail of the d*S* distribution (supplementary figs. 1 and 2, Supplementary Material online).

The most striking difference between the murine and the gerbil species is the excess of genes with high d*S*_WS_ in the two gerbil species (fig. 3*B* and supplementary fig. 3, Supplementary Material online). Respectively 4.1% and 4.4% of genes of *P. obesus* and *M. unguiculatus* were outliers in d*S*_WS_ (360 and 387 out of 8,809, respectively; supplementary table 1, Supplementary Material online), of which 323 were outliers in both species (fig. 3*C*). By comparison, only 0.7% of genes in *M. musculus* and *R. norvegicus* were outliers in d*S*_WS_ (64 and 65 out of 8,809, respectively). Thus, we conclude that the genomes of gerbil species include a large number of genes with outlying d*S*_WS_ values, located outside the known high-GC region.

**Fig. 4.**
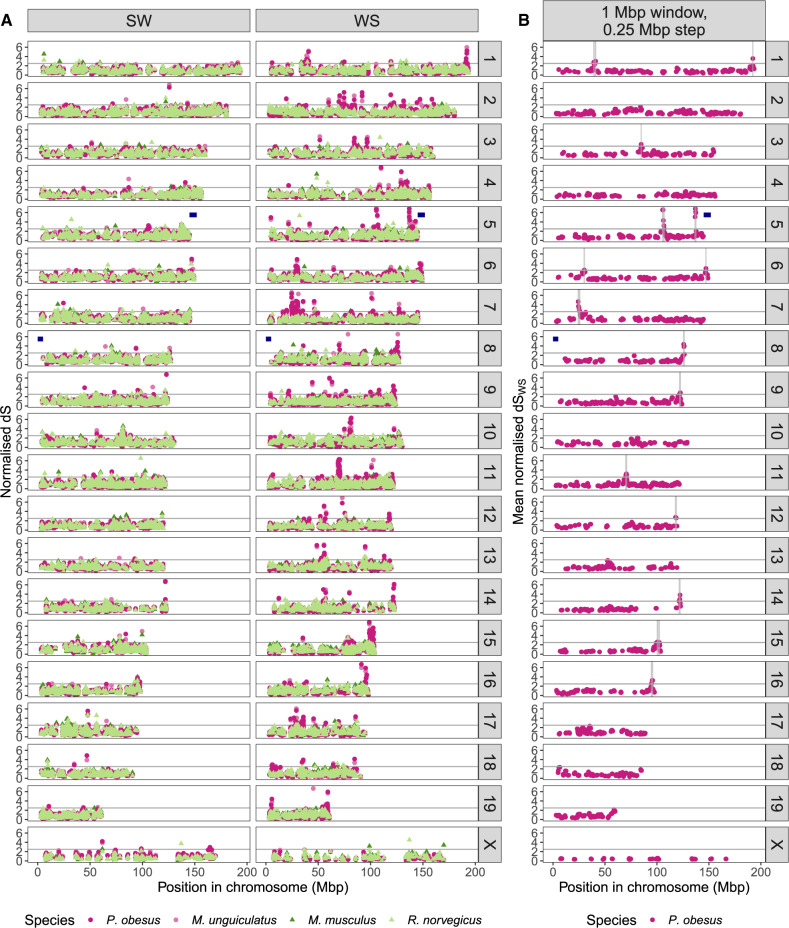
Clustering of outliers in the weak-to-strong d*S*. (*A*) Normalized rate of synonymous substitution (d*S*) for two mutational categories, strong-to-weak (SW) and weak-to-strong (WS), for 8,809 genes; (*B*) average normalized weak-to-strong d*S* (d*S*_WS_) in sliding windows of 1 Mb with a step of 0.25 Mb, showing only windows with >3 genes, and with outlying regions marked with a vertical gray bar. The genes are mapped by row to the chromosomes of the *Mus musculus* reference genome assembly. The horizontal gray line represents the d*S* threshold chosen to define outlier genes (d*S* >2.5 times the average for the respective species and mutational category). The dark blue horizontal bars represent the location of the previously known high-GC region.

We then asked whether these genes also have a higher d*S* value for the other mutational categories. Comparing the values of d*S*_SW_, d*S*_SS_, and d*S*_WW_ between outlier and nonoutlier genes shows that, in all species, the outlier genes have higher substitution rates than nonoutlier genes for these three mutational categories (one-tailed Wilcoxon rank-sum test, *P* < 0.05 for all categories in all species, supplementary table 2 and fig. 4, Supplementary Material online). It is important to note, however, that this difference is not extreme: The genes with outlying d*S*_WS_ did not have a disproportionately large number of genes that also had an outlying value for the other three mutational categories (chi-squared test for each species, *P* > 0.05; supplementary fig. 4, Supplementary Material online). In other words, although the genes that are classed as outliers based on d*S*_WS_ have on average higher values for the other three mutational categories, some other genes in the genome can be classed as outliers based on these three mutational categories. The difference between outliers and nonoutliers was least strong for d*S*_SW_ than for d*S*_WW_ or d*S*_SS_ (supplementary table 2, Supplementary Material online). For instance, in *P. obesus* the difference in median normalized d*S*_SW_ between outliers and nonoutliers was only 0.14, compared with a difference in median normalized d*S*_SS_ of 0.77.

Despite the average increase in all mutational categories in the genes with outlying d*S*_WS_, are these genes affected by a GC skew? We found that, in our four focal species, >90% of genes with outlying d*S*_WS_ had higher d*S*_WS_ than its opposing rate, d*S*_SW_ (99% in *P. obesus*, 98% in *M. unguiculatus*, 95% in *M. musculus*, and 92% in *R. norvegicus*; fig. 3*B*). This effect is particularly striking for the two gerbil species. As seen in figure 3*B*, the relationship between d*S*_WS_ and d*S*_SW_ in these two species has a “chimney” shape, indicating a large excess of d*S*_WS_. In other words, the two gerbil species had a large number of genes with an outlying d*S*_WS_, of which virtually all had higher d*S*_WS_ than its opposing rate. We thus conclude that gerbils are affected by GC-skewed evolution in protein-coding genes outside the known high-GC region.

Is this GC skew large enough to have affected the GC-content of gerbil genes since their divergence from the murines? To study this, we measured GC3 of the representative sequences for the four focal species in our analysis for each of the 8,809 groups of orthologous genes (excluding genes for which the coding sequence annotation length was not a multiple of three: 12 genes in *M. musculus*, 5 in *M. unguiculatus*, 2 in *R. norvegicus*, and 0 in *P. obesus*). The GC3 distributions of each of the four species were different from one another (Kruskal–Wallis rank sum test, *χ*^2^ = 136.02, df = 3, *P* < 1  10^−28^; table 1; supplementary fig. 5, Supplementary Material online). Pairwise comparisons between the species show a significant difference only between the murine and the gerbil species (Dunn’s Kruskal–Wallis multiple comparisons with the Benjamini–Hochberg multiple testing correction, *P* < 0.001), with no difference between *M. musculus* and *R. norvegicus* or between *P. obesus* and *M. unguiculatus*. The difference between the two clades is seen as bias toward high GC3 in the gerbil species (supplementary fig. 6, Supplementary Material online). Is this bias explained by the process of GC-skewed substitution seen in the gerbil genomes? Figure 3*D* shows that the genes with an outlying value of d*S*_WS_ in *P. obesus* are also those with the largest difference in GC3 between this species and *M. musculus*, a result that is mirrored in all gerbil-murine comparisons (supplementary fig. 7, Supplementary Material online). Thus, we conclude that the genes in the gerbil lineage underwent a GC-skewed substitution process that has increased their GC3 relative to their orthologs in the murine lineage.

**Table 1.** GC-Content in the Third-Codon Position (GC3) for the Four Species in 8,809 Orthologous Groups.

Species	Mean GC3 (%)	SD (%)
*Psammomys obesus*	62.46	12.64
*Meriones unguiculatus*	62.85	12.88
*Mus musculus*	61.28	10.73
*Rattus norvegicus*	61.19	10.45

Genes under GC-skewed evolution in other clades have been documented to have a relatively high load of deleterious mutations (Berglund et al. 2009; Galtier et al. 2009; Necşulea et al. 2011). We determined whether the outliers in d*S*_WS_ are affected by putatively deleterious substitutions by testing whether these genes have an elevated rate of nonsynonymous changes relative to the other genes. For both gerbil species, the group of outlier genes had higher d*N*_WS_, d*N*_SS_, and d*N*_WW_—but not d*N*_SW_—than the nonoutlier genes (one-tailed Wilcoxon rank sum test, *P* < 0.05 for each significant comparison, supplementary table 3 and fig. 8, Supplementary Material online). The difference was strongest for the WS mutational category, normalized d*N*_WS_ having a median value of 1.59 and 1.54 for the outliers of *P. obesus* and *M. unguiculatus*, compared with 0.65 and 0.64, respectively, for the nonoutliers of each species. These results suggest that the outlier genes have a higher load of deleterious mutations than nonoutlier genes. Nevertheless, it is important to note that the difference between the two groups of genes was modest for all mutational categories, with a large overlap in their ranges (supplementary fig. 8, Supplementary Material online).

### GC-Skewed Genes Are Organized in Clusters

Above, we have shown that >300 of the genes in the 8,809 orthology groups have an outlying d*S*_WS_ in gerbils (fig. 3*C*), representative of a GC-skewing process specific to the gerbil lineage. An important question is whether these genes are positioned randomly across the genome, or whether they are organized into clusters in a similar way to the previously described high-GC region of *P. obesus*. Because gerbil genomes have not been assembled to sufficient contiguity to test this directly, we mapped each gene to the mouse reference assembly. In figure 4*A*, it can be clearly seen that genes with high d*S*_WS_ in the gerbil species tend to be placed in close proximity to each other, forming peaks in the spatial distribution (see supplementary fig. 9, Supplementary Material online for the other mutational categories and supplementary fig. 10, Supplementary Material online for the distribution of GC12 and GC3). These groups of genes form peaks present on every chromosome except for the X chromosome.

We performed permutations to test whether outlier genes in figure 3*C* are placed closer to each other than what would be expected if they were distributed randomly. The observed distance between d*S*_WS_ outliers (5.39 Mb in *P. obesus* and 5.14 Mb in *M. unguiculatus*) was smaller than most permutations (*P* = 1  10^−6^ for *P. obesus*, *P* = 4  10^−6^ for *M. unguiculatus*, supplementary table 4 and fig. 11, Supplementary Material online). The very few outlier genes of *M. musculus*, but not of *R. norvegicus*, also had a smaller distance between each other than most permutations, although not as extremely as in the two gerbil species (*P* = 0.015 for *M. musculus*, *P* = 0.36 for *R. norvegicus*, supplementary table 4 and fig. 11, Supplementary Material online). Consistent with this observation, the two gerbil species had more outlier genes than expected inside “islands,” defined as runs of two or more neighboring outlier genes (42% of the outliers for *P. obesus*; 42% of the outliers for *M. unguiculatus*; *P* ≈ 0 for both species; supplementary table 4 and fig. 11, Supplementary Material online). The fact the outlier genes tend to be organized into clusters also implies that the gerbil species have large tracts of chromosome without any outliers in the GC skew process (fig. 4*A*).

How many regions in the genome include clusters of outlying genes? The difficulty of defining the exact boundaries of such regions is that runs of outlier genes are interspersed by genes with low d*S*_WS_. We divided the genome into sliding windows of 1 Mb overlapping with a step of 0.25 Mb. We identified any window where the median d*S*_SW_ is >2.5 times the average d*S*_SW_ for each of the species, which we defined as “outlying regions.” Overlapping windows that passed this threshold were collapsed into single regions. We found 17 such regions each for *P. obesus* and for *M. unguiculatus*, respectively containing 34% and 30% of the outlying genes of each species (fig. 4*B*;supplementary fig. 12 and table 5, Supplementary Material online). By comparison, we found no regions for either *M. musculus* or *R. norvegicus*. With a smaller window size (0.5 Mb with a 0.1 Mb step), we found a qualitatively similar result, although the gerbil species had a larger number of outlying regions (supplementary fig. 13 and table 5, Supplementary Material online).

Studies of GC-biased substitutions since the divergence between human and chimpanzee have found that GC-biased substitutions occur at a higher density in subtelomeric regions in these species (Dreszer et al. 2007; Auton et al. 2012; Capra et al. 2013; Munch et al. 2014). To test whether a similar pattern is seen in the gerbil genomes, we determined whether there is an excess of d*S*_WS_ outliers mapped to the 5 Mb region at the start and at the end of each chromosome in the mouse reference assembly. We found that ∼20% of the outlier genes in *P. obesus* and *M. unguiculatus* are located in these subtelomeric regions, compared with only 6% of the nonoutlier genes (*χ*^2^ test, *P* < 0.05, supplementary table 6, Supplementary Material online). Indeed, for *P. obesus* and *M. unguiculatus*, the inferred subtelomeric regions included respectively 9 of the 17 and 8 of the 17 “outlying regions” identified in the sliding window analysis above (supplementary table 7, Supplementary Material online). However, the gerbil and murid lineages are known to have different karyotypes, so it is likely that not all subtelomeric regions in the gerbil species are subtelomeric in mouse and *vice versa*. To place the outlier genes onto a gerbil karyotype, we used the genetic map of *M. unguiculatus* produced by Brekke et al (2019). This map includes only 1,720 out of the 8,809 (20%) sets of orthologous genes used in our analyses (and 70 out of 387 of those that were outliers in d*S*_WS_ in *M. unguiculatus*, 18%), the remaining being located in unmapped genomic scaffolds. Thus, we could not assess whether the subtelomeric regions of *M. unguiculatus* are represented in the genetic map and we were therefore unable to test whether these regions are enriched for d*S*_WS_ outliers. Nevertheless, 52 of the 70 genes present in the genetic map that were d*S*_WS_ outliers in *M. unguiculatus* were not mapped to the first or last 2.4 cM of any of the linkage groups (i.e., they are very likely not subtelomeric). This set of genes includes all 10 outlier genes located in the subtelomeric regions of the *M. musculus* assembly that could be placed on the *M. unguiculatus* genetic map. Thus, we conclude that there are d*S*_WS_ outliers located outside subtelomeric regions, including some genes that are subtelomeric in *M. musculus*.

### Many of the GC-Skewed Genes Have the Highest GC3 in Their Gene Families

Above, we have shown that gerbil species carry many genes with outlying GC-skewed substitution rates relative to their orthologs in two other rodents. Despite our observation that these outlying genes tend to have higher GC-content than their orthologs in the murine species, it is not clear whether GC-skewed evolution of these genes has increased their GC-content compared with a wider phylogenetic level. To measure the extent of GC accumulation in gerbils, we compared the gerbil sequences in each orthologous group with the wider gene family to which the group belongs. For this, we assigned each orthologous group to a Hierarchical Orthology Group (HOG), defined as all orthologous and paralogous genes with a single common ancestor, as identified in the OMA database (Altenhoff et al. 2013, 2018). We assigned 6,735 of the orthologous groups to HOGs. For each, we determined the rank of the GC3 value for the two gerbil and the two murine species relative to the sequences in the deuterostome species in each HOG (we excluded *M. musculus* and *R. norvegicus* from the HOG database to avoid double-counting). We found that gerbil species had an enrichment of genes with a high-GC3 rank in their HOGs relative to the murine species. For instance, 183 and 208 of the genes in *P. obesus* and *M. unguiculatus*, respectively, were in the top three highest GC3 of their HOGs (2.7% and 3.1% of 6,735 genes), compared with only 58 and 65 in *M. musculus* and *R. norvegicus*, respectively (0.9% and 1.0% of 6,735 genes; *χ*^2^ test, *χ*^2^ = 145.13, df = 3, *P* < 1 × 10^−30^). For all species, genes classed as d*S*_WS_ outliers had a higher rank position (high GC3 to low GC3) than those not classed as such (one-sided Wilcoxon rank sum test for each species, *P* < 0.05, supplementary fig. 14, Supplementary Material online). This pattern was most pronounced in the gerbil species. For instance, 90 genes of the 183 genes in the top three highest GC3 of their HOGs (49%) in *P. obesus* were classed as d*S*_WS_ outliers, contrasting to only 6 of 58 (10%) in *M. musculus* (Fisher’s exact test comparing the four species, *P* < 10^−19^; supplementary table 8, Supplementary Material online). In summary, we found that GC-skewed evolution in the d*S*_WS_ outliers of the gerbil species has allowed many of these genes to evolve a higher GC3 than most of their homologues across the animal kingdom.

## Discussion

Previous work identified a genomic region containing genes with very high GC-content in the genome of a gerbil (Hargreaves et al. 2017). The extreme nature of this region and its relatively recent origin in the gerbil lineage raises important questions about the nature of GC skew in genomes, its mechanism of origin, its phylogenetic distribution and its impact on the evolution of genes. Here, we explore these issues and make some surprising findings concerning the evolution of GC-content in animal genomes.

Our study identifies two patterns of GC skew in gerbil genomes. The first type of skew is seen in the previously described high-GC region (Hargreaves et al. 2017). We find this region has been affected by extremely high substitution rates of all types (fig. 1). For *P. obesus* and *M. unguiculatus*, all but one gene in this region had substitution rates within the top percentile of the genome-wide distribution, as measured from the 8,809 groups of orthologous genes in the rest of the genome (supplementary table 9 and supplementary fig. 15, Supplementary Material online). In the gerbil species, all genes in the region had a higher weak-to-strong than strong-to-weak substitution rate, consistent with an overall dramatic increase in GC-content. These results are not restricted to “silent” sites but are also evident in mutations that change amino acids: d*N* values reveal evidence for considerable evolution at the amino-acid level driven by GC skew (fig. 2). A second type of GC skew is observed across the rest of the genome, where we see at least 17 large clusters of genes with outlying values of weak-to-strong d*S* (figs. 3 and 4). These values are considerably higher than generally seen in mouse and rat (fig. 3*B*), yet they are not as extreme as seen in the previously described region. These genome outliers show only a modest increase in the other three substitution rate categories for d*S*, and a modest increase in the weak-to-strong d*N*. The consequence in these genomic regions is a small increase in GC-content at particular regions.

A hypothesis explaining the GC-skewed evolution of both categories of high-GC genes is a drastic increase in the efficacy of gBGC in the gerbil evolutionary lineage. It is important to note that any such increase must have evolved in a relatively short period of time. The Murinae diverged from the clade that includes the gerbils 20.6–22.5 Ma (Steppan et al. 2004). This clade includes Deomyinae, which is the sister clade to the Gerbillinae (the gerbils), the two having diverged between 17.6 and 20 Ma (Steppan et al. 2004). Previous work has shown that the *Pdx1* gene of a species in Deomyinae, *Acomys cahirinus*, does not have a higher GC-content than mouse or rat (Hargreaves et al. 2017), suggesting that the GC skew we observed evolved after the split between Gerbillinae and Deomyinae.

What could have caused an increase in the action of gBGC in the gerbil lineage? The strength of gBGC results from the interaction between a number of different factors: Effective population size (Ne), the length of conversion tracts, the magnitude of the GC bias within each conversion event, and the recombination rate (Glémin et al. 2015; Galtier et al. 2018). Regarding Ne, large populations are expected to be subject to more effective gBGC at neutral sites (Romiguier et al. 2010; Weber et al. 2014; Glémin et al. 2015; Borges et al. 2019), although it is difficult to speculate whether murine and gerbil species have different demographic histories. The second factor that is expected to increase gBGC is the conversion tract length: That is, the length of the single-stranded DNA sequence that invades from one homologous chromosome into another at meiosis. Although studies have reported conversion tracts with a range of different lengths (from ∼30 to ∼1,000 nucleotides in humans and mice) (Cole et al. 2014; Williams et al. 2015; Halldorsson et al. 2016; Li et al. 2019), it is currently unclear whether this variation is a methodological artifact. Therefore, the hypothesis that systematic differences in tract length between species or between different chromosomal regions could explain variation in gBGC is currently speculative. The third factor is the magnitude or strength of GC bias in a noncrossover. Again, it is unknown whether the magnitude of GC bias can differ between species, although recent studies have shown that both humans and mice have a similar conversion bias (∼68%) in noncrossover events at heterozygous sites (Williams et al. 2015; Li et al. 2019).

The fourth factor affecting the strength of gBGC is the rate of recombination, which, as explained in the Introduction, has been shown to mediate differences in GC-content between species. At the local level, the rate of recombination is mediated by the rate of double-strand breaks, which are thought to be controlled by two pathways (Brick et al. 2012, 2018). The first involves the zinc-finger protein PRDM9, which controls the localization of double-strand breaks by mobilizing recombination machinery (specifically the protein SPO11) to PRDM9-binding sites across each chromosome. These sites undergo rapid turnover (Myers et al. 2010; Brick et al. 2012; Smagulova et al. 2016; Latrille et al. 2017), creating transient hotspots of recombination. The second pathway, which is preferentially used by females in mice, involves the mobilization of the recombination machinery to specific histone modification marks near promoters in gene-rich regions of the genome (Brick et al. 2012, 2018). In theory, a change in either of the pathways could affect the process of gBGC. An example is seen in canids, where loss of the *Prdm9* gene has caused recombination hotspots to be focused in certain small regions of the genome, which are consequently affected by strong gBGC (Axelsson et al. 2012). Additionally, changes in the action of gBGC can be caused by changes to the larger-scale mechanisms that control the distribution of the recombination hotspots (Coop and Przeworski 2007; Stapley et al. 2017). For example, studies of the human and chimpanzee genomes have shown that average recombination rates—and the strength of gBGC—are conserved between these two species over large genomic windows, with recombination rates being particularly high in subtelomeric regions (Dreszer et al. 2007; Auton et al. 2012; Capra et al. 2013; Munch et al. 2014). The implication is that species with similar recombination landscapes are affected by gBGC in similar regions, whereas changes to recombination landscapes are expected to cause divergence in the effect of gBGC. This hypothesis is supported by studies of GC-content at broad evolutionary levels, which show a inverse correlation between GC-content and chromosome size in several metazoan clades (Romiguier et al. 2010; Galtier et al. 2018). Given that smaller chromosomes tend to have higher rates of recombination, these studies support the idea that karyotype is a key determinant of the location of recombination hotspots and of the strength of gBGC.

Our study presents several lines of evidence supporting the hypothesis that changes to the recombination rate in the gerbil lineage caused the GC skew we observed. First, the outliers in weak-to-strong d*S* were affected by a modest increase of d*S* for the three other mutational categories, which could reflect the mutagenic effect of recombination itself, a phenomenon also seen in birds (Bolívar et al. 2016; Rousselle et al. 2019) and mammals (Pratto et al. 2014; Arbeithuber et al. 2015; Smith et al. 2018; Rousselle et al. 2019). Second, the weak-to-strong outliers were also affected by a higher rate of nonsynonymous substitutions, consistent with a relatively high load of deleterious mutations as seen in genes affected by gBGC in other species (Berglund et al. 2009; Galtier et al. 2009; Necşulea et al. 2011). Third, the gerbil weak-to-strong outliers were clustered in the genome, as expected if they were caused by clusters of recombination hotspots (Dreszer et al. 2007; Auton et al. 2012; Capra et al. 2013; Munch et al. 2014). Fourth, comparing the gerbil and the murine species gene-by-gene and window-by-window showed only a low level of correlation in the weak-to-strong substitution rate (supplementary figs. 16 and 17, Supplementary Material online). This result suggests that the recombination landscapes of gerbil and murine species are divergent, and is consistent with the low levels of correlation in the recombination rate found even within murine species (Jensen-Seaman et al. 2004). The fact that there were very few weak-to-strong d*S* outliers in the genomes of *M. musculus* and *R. norvegicus* suggests that the gerbil species have recombination rates higher than the murine species. It is important to note that our data do not allow us to infer whether the regions with putatively high recombination rates in gerbils have much higher rates than similar regions in mammals other than mouse and rat. However, the fact that the GC-content of the outlier genes had a high rank compared with their wider gene families in deuterostomes suggests this is likely.

Interestingly, the recombination rate of subtelomeric regions of *M. musculus* and *R. norvegicus* is known not to be as high relative to the rest of the genome as seen in primates (Jensen-Seaman et al. 2004). This may explain how approximately half of the weak-to-strong outliers in gerbils can map to subtelomeric regions in the *M. musculus* genome assembly yet not be outliers in this species. Nevertheless, high rates of karyotypic evolution have been documented in muroid rodents (Ferguson-Smith and Trifonov 2007), with gerbil species differing in chromosome number relative to mice and carrying interstitial telomere sites, evidence of several large structural changes (de la Fuente et al. 2014). This level of karyotypic change in murids implies that subtelomeric regions of the mouse genome may not be subtelomeric in gerbils, and *vice versa*. Future work would therefore require the production of more highly contiguous gerbil genome assemblies such that regions of recombination and GC skew can be mapped at a chromosomal level. In addition to karyotypic changes, changes to chromatin states during meiosis can also modulate local rates of double-strand breaks (Saccone et al. 2002). For instance, genes and transposable elements that are active during meiosis are marked with the histone modification that initiates double-strand breaks in the PRDM9-independent pathway, in a process controlled by DNA methylation (Zamudio et al. 2015). The clusters of GC-skewed outlier genes could thus represent regions of the chromosome with high accessibility to recombination machinery. It has been suggested that the previously known high-GC region is exceptionally repetitive (Hargreaves et al. 2017). A tantalizing hypothesis to explain the extreme substitution rates that we observed in this region is that its chromatin state is particularly accessible during meiosis.

In summary, we have shown that the genomes of gerbil species carry protein-coding genes with outlying levels of GC-skewed substitution. We propose that changes to the recombination landscape in this lineage seem a likely explanation for the existence of multiple regions of GC skew. Studying the mechanisms that control the large scale evolution of recombination rates would give us a better understanding of the evolution of GC-content, including the evolution and maintenance of isochore structures in vertebrates (Costantini et al. 2009). We expect that the GC-skewed evolution that we characterized in the gerbil lineage can be used as a model for the study of these processes.

## Materials and Methods

### Transcriptomic Sequencing and Assembly

Many of the genes in the high-GC region of the fat sand rat *P. obesus* are not present in the genome assembly because of biases in the sequencing and assembly of high-GC sequences. Previously, Hargreaves et al (2017) determined the sequence of several of these genes by producing transcriptome assemblies of several tissues. We repeated this analysis for three additional species, *M. unguiculatus*, *M. Shawi*, and *M. lybicus*. For each species, we sequenced the transcriptomes of kidney and liver (all species) and duodenum (*M. unguiculatus* and *M. libycus* only). We also sequenced pancreas RNA of *M. libycus* and generated additional transcriptomic data for the duodenum, kidney, and testis of *P. obesus*. Animal handling was in accordance with European Union and UK Home Office animal care regulations, and approved by local animal welfare and ethical review boards. Tissues were snap-frozen on dry ice except for pancreas, which was homogenized immediately in TRIreagent; total RNA was extracted and purified by using TRIreagent followed by DNAse I treatment and reprecipitation. mRNA was prepared for sequencing using the TruSeq stranded mRNA sample preparation kit (Illumina) with polyA selection. All libraries were then pooled and sequenced using 75-bp paired-end reads across two lanes of the Illumina HiSeq4000 platform. The quality of all sequencing data was assessed using FastQC (www.bioinformatics.babraham.ac.uk/projects/fastqc/). Adapter contamination was removed from raw sequencing reads using Trimmomatic (Bolger et al. 2014) and subsequently quality trimmed using Sickle (Joshi and Fass 2011). Reads were then pooled per species and assembled with Trinity (Grabherr et al. 2011) using default parameters. The newly generated data for *P. obesus* were combined with previously generated transcriptomic data for pancreatic islets and liver (Hargreaves et al. 2017) prior to assembly. Putative transcript coding sequences were first identified using BLAST+ (Camacho et al. 2009) using query amino acid sequences corresponding to orthologous genes located within the high-GC region from three rodent species (mouse, rat, and sand rat). Sequences for mouse and rat were obtained from either Ensembl or GenBank, and *P. obesus* sequences from previously generated sequence data (Hargreaves et al. 2017). Putative transcripts were reciprocally aligned with BLAST against the NCBI nr database to confirm the putative transcript ID. Coding sequences were then annotated manually to ensure that the correct open reading frame was recovered.

### Orthology Identification

We identified the longest transcript of predicted gene sequences from Ensembl (release 95) in 12 rodents and *H. sapiens* (fig. 3*A*; accession IDs in supplementary table 10, Supplementary Material online). From these, we identified groups of orthologous sequences (orthogroups) using Orthofinder version 2.2.7 (Emms and Kelly 2015, 2019). We performed separate Orthofinder runs for the high-GC region data set (for which we did not include any gerbil species) and for the whole-genome data set (for which we included *P. obesus* and *M. unguiculatus*). Orthofinder was run using default parameters, the diamond aligner version 0.9.21 (Buchfink et al. 2015) and the species tree topology shown in figure 3*A*. We retrieved the orthogroups that had a single copy in all species, with the exception of a maximum of two species with zero or more than one representative gene (in which case these species were removed from the orthogroup). For the whole-genome data set, we did not allow this exception on the species in the murid lineage (Muridae and Gerbillinae, fig. 1) or on *H. sapiens*. For the high-GC region data set, we only analyzed 27 orthogroups that included genes known to be in the high-GC region and a representative sequence in at least one gerbil species, to which we added the manually annotated gerbil sequences.

### Alignment

We used the alignSequences program of MACSE version 2.03 (Ranwez et al. 2018) with default parameters to align the nucleotide and amino acid sequences in each of these orthogroups. We recoded the alignment for downstream use with the exportAlignment of MACSE, with options “-codonForInternalStop NNN -codonForExternalFS --- -codonForInternalFS ---”. We removed nonhomologous sequences from each amino acid alignment using HmmCleaner.pl version 0.180750 (Arnauld di Franco, available at https://metacpan.org/pod/HmmCleaner.pl, last accessed March 30, 2020) with default parameters. We transferred the amino acid filter to the nucleotide alignments using the reportMaskAA2NT program of MACSE with parameters “-min_NT_to_keep_seq 30 -mask_AA $-min_seq_to_keep_site 4 -min_percent_NT_at_ends 0.3 -dist_isolate_AA 3 -min_homology_to_keep_seq 0.3 -min_internal_homology_to_keep_seq 0.5”, which performs additional alignment cleaning of nonhomologous sequences.

In the whole-genome data set, many alignments include partial gene sequences (supplementary fig. 18, Supplementary Material online). To remove the worst affected alignments, we first eliminated those for which the longest gene was <400 bp. For each alignment, we then masked any species sequence with gaps representing >40% of the nongap size of the longest sequence in the alignment. After these filters, we allowed a maximum of two species to have a masked or missing gene (except for *H. sapiens, M. musculus*, *R. norvegicus*, *P. obesus*, and *M. unguiculatus*). After filtering, the whole-genome data set included 8,815 aligned orthogroups. We removed the six orthogroups with genes located in the known high-GC region, resulting in 8,809 orthogroups (the remaining genes in the known high-GC region were either not represented in the genome assemblies of *P. obesus* or *M. unguiculatus*, or otherwise not represented in the orthogroups).

### Substitution Rates

We estimated rates of synonymous and nonsynonymous substitution (d*S* and d*N*, respectively) for different mutational categories for each nucleotide alignment using the BppSuite (Guéguen et al. 2013). For each alignment, we trimmed the tree in figure 1*A* to include only the species in the alignment. We used the BppML subprogram version 2.3.1 of BppSuite to optimize branch lengths for each alignment, using the YN98 (F3X4) model (Yang and Nielsen 1998) and the parameters available online (script 1, Pracana and Hargreaves 2019). We then estimated d*S* and d*N* for different mutational categories (weak-to-strong, strong-to-weak, weak-to-weak, and strong-to-strong) using BppML subprogram MapNH version 1.1.1 (Romiguier et al. 2012) with the parameter “map.type = Combination(reg1 = d*N*d*S*, reg2 = SW)” (script 2, Pracana and Hargreaves 2019). For the high-GC region data set, the program could not be run successfully for five of the alignments, as the gerbil sequences were too short. For each of the two data sets, we retrieved the d*S* and d*N* value for *M. musculus*, *R. norvegicus* and the gerbil species by summing, for each species, the branch lengths from the Muridae node to the tree tip. We used an R script to measure GC, GC12, and GC3 for the original nontrimmed sequence of each species for each alignment.

### d*S*_WS_ Outlier Clusters

We performed 1 million permutations for each of the focal species to test whether d*S*_WS_ outliers are closer to each other in the *M. musculus* reference genome assembly GRCm38.p6 (Ensembl release 95) than would be expected if they were randomly distributed among the genes in our analysis. In each permutation, we assigned the number of d*S*_WS_ outliers in the species to random genes. We then measured the inferred distance between these genes and the number that fell in runs of more than two neighboring genes. Additionally, we identified genomic regions for each species where genes have high average d*S*_WS_. For this, we divided the genome into overlapping sliding windows (1 Mb with a 0.25-Mb step and 0.25 Mb with a 0.1-Mb step along the mouse reference assembly) and determined whether the average normalized d*S*_WS_ among the genes of each species was >2.5 times the average d*S*_WS_ among the 8,809 genes for that species, ignoring windows with less than four mapped groups of orthologous genes. We defined subtelomeric regions as those located within 5 Mb of the start and end of each *M. musculus* chromosome. To position genes onto the *M. unguiculatus* linkage groups, we used the genetic map produced by Brekke et al (2019), which gives the average centimorgan position of each mapped scaffold in the *M. unguiculatus* genome assembly. We considered any scaffold mapped within 2.85 cM of linkage group starts or ends as subtelomeric, a threshold approximately equivalent to 5 Mb considering the mouse average recombination rate of 0.57 cM/Mb (Cox et al. 2009).

### Hierarchical Orthology Groups

We downloaded the HOG database (version June 2018) from OMA orthology database (Altenhoff et al. 2013, 2018). For each of the orthogroups (above), we used the mouse transcript ID to assign the orthogroup to a HOG. For each of the resulting 6,736 HOGs, we retrieved the sequences of all genes for all represented deuterostome species, excluding any sequences from *M. musculus* and *R. norvegicus*, and used an R script to measure their GC3. For each orthogroup, we then independently compared the rank of the GC3 for each of the four focal species (*M. musculus*, *R. norvegicus*, *P. obesus*, and *M. unguiculatus*) relative to the HOG GC3 measurements.

### Data Availability

Data sets and BppML parameters used in this study are available online at the Oxford University Research Archive (data sets 1–10 and scripts 1 and 2; Pracana and Hargreaves 2019):

- Data set 1: Assembled transcriptome sequences for the high-GC region.
- Data set 2: Rate and GC-content measurements for the high-GC region.
- Data set 3: Predicted coding sequences for *P. obesus.*
- Data set 4: Predicted protein sequences for *P. obesus.*
- Data set 5: Transcript ID of each sequence in the 8,809 groups of orthologous genes.
- Data set 6: Rate measurements for the 8,809 groups of orthologous genes.
- Data set 7: Normalized rate measurements for the 8,809 groups of orthologous genes.
- Data set 8: GC-content measurements for the 8,809 groups of orthologous genes.
- Data set 9: Sliding-window measurements.
- Data set 10: GC ranking of the focal species relative to their HOGs.

## Supplementary Material

msaa072_Supplementary_Figures_Tables

## References

[msaa072-B1] Altenhoff AM, Gil M, Gonnet GH, Dessimoz C. 2013 Inferring hierarchical orthologous groups from orthologous gene pairs. PLoS One 8(1):e53786. 23342000 10.1371/journal.pone.0053786 PMC3544860

[msaa072-B2] Altenhoff AM, Glover NM, Train C-M, Kaleb K, Warwick Vesztrocy A, Dylus D, de Farias TM, Zile K, Stevenson C, Long J, et al 2018 The OMA orthology database in 2018: retrieving evolutionary relationships among all domains of life through richer web and programmatic interfaces. Nucleic Acids Res. 46(D1):D477–D485. 29106550 10.1093/nar/gkx1019 PMC5753216

[msaa072-B3] Arbeithuber B, Betancourt AJ, Ebner T, Tiemann-Boege I. 2015 Crossovers are associated with mutation and biased gene conversion at recombination hotspots. Proc Natl Acad Sci USA. 112(7):2109–2114. 25646453 10.1073/pnas.1416622112 PMC4343121

[msaa072-B4] Arnheim N, Calabrese P, Tiemann-Boege I. 2007 Mammalian meiotic recombination hot spots. Annu Rev Genet. 41(1):369–399. 18076329 10.1146/annurev.genet.41.110306.130301

[msaa072-B5] Auton A, Fledel-Alon A, Pfeifer S, Venn O, Ségurel L, Street T, Leffler EM, Bowden R, Aneas I, Broxholme J, et al 2012 A fine-scale chimpanzee genetic map from population sequencing. Science 336(6078):193–198. 22422862 10.1126/science.1216872 PMC3532813

[msaa072-B6] Axelsson E, Webster MT, Ratnakumar A, Ponting CP, Lindblad-Toh K, The LUPA Consortium 2012 Death of PRDM9 coincides with stabilization of the recombination landscape in the dog genome. Genome Res. 22(1):51–63. 22006216 10.1101/gr.124123.111 PMC3246206

[msaa072-B7] Berglund J, Pollard KS, Webster MT. 2009 Hotspots of biased nucleotide substitutions in human genes. PLoS Biol. 7(1):e1000026. 10.1371/journal.pbio.1000026 PMC2631073 19175294

[msaa072-B8] Bolger AM, Lohse M, Usadel B. 2014 Trimmomatic: a flexible trimmer for Illumina sequence data. Bioinformatics 30(15):2114–2120. 24695404 10.1093/bioinformatics/btu170 PMC4103590

[msaa072-B9] Bolívar P, Guéguen L, Duret L, Ellegren H, Mugal CF. 2019 GC-biased gene conversion conceals the prediction of the nearly neutral theory in avian genomes. Genome Biol. 20(1):5. 10.1186/s13059-018-1613-z PMC6322265 30616647

[msaa072-B10] Bolívar P, Mugal CF, Nater A, Ellegren H. 2016 Recombination rate variation modulates gene sequence evolution mainly via GC-biased gene conversion, not Hill–Robertson interference, in an avian system. Mol Biol Evol. 33(1):216–227. 26446902 10.1093/molbev/msv214 PMC4693978

[msaa072-B11] Borges R, Szöllősi GJ, Kosiol C. 2019 Quantifying GC-biased gene conversion in great ape genomes using polymorphism-aware models. Genetics 212(4):1321–1336. 31147380 10.1534/genetics.119.302074 PMC6707462

[msaa072-B12] Botero-Castro F, Figuet E, Tilak M-K, Nabholz B, Galtier N. 2017 Avian genomes revisited: hidden genes uncovered and the rates versus traits paradox in birds. Mol Biol Evol. 34(12):3123–3131. 28962031 10.1093/molbev/msx236

[msaa072-B13] Brekke TD, Supriya S, Denver MG, Thom A, Steele KA, Mulley JF. 2019 A high-density genetic map and molecular sex-typing assay for gerbils. Mamm Genome. 30(3–4):63–70. 30972478 10.1007/s00335-019-09799-z PMC6491409

[msaa072-B14] Brick K, Smagulova F, Khil P, Camerini-Otero RD, Petukhova GV. 2012 Genetic recombination is directed away from functional genomic elements in mice. Nature 485(7400):642–645. 22660327 10.1038/nature11089 PMC3367396

[msaa072-B15] Brick K, Thibault-Sennett S, Smagulova F, Lam K-W, Pu Y, Pratto F, Camerini-Otero RD, Petukhova GV. 2018 Extensive sex differences at the initiation of genetic recombination. Nature 561(7723):338–342. 30185906 10.1038/s41586-018-0492-5 PMC6364566

[msaa072-B16] Buchfink B, Xie C, Huson DH. 2015 Fast and sensitive protein alignment using DIAMOND. Nat Methods. 12(1):59–60. 25402007 10.1038/nmeth.3176

[msaa072-B17] Camacho C, Coulouris G, Avagyan V, Ma N, Papadopoulos J, Bealer K, Madden TL. 2009 BLAST+: architecture and applications. BMC Bioinformatics 10:421. 10.1186/1471-2105-10-421 PMC2803857 20003500

[msaa072-B18] Capra JA, Hubisz MJ, Kostka D, Pollard KS, Siepel A. 2013 A model-based analysis of GC-biased gene conversion in the human and chimpanzee genomes. PLoS Genet. 9(8):e1003684. 23966869 10.1371/journal.pgen.1003684 PMC3744432

[msaa072-B19] Chen Y-C, Liu T, Yu C-H, Chiang T-Y, Hwang C-C. 2013 Effects of GC bias in next-generation-sequencing data on *de novo* genome assembly. PLoS One 8(4):e62856. 23638157 10.1371/journal.pone.0062856 PMC3639258

[msaa072-B20] Cole F, Baudat F, Grey C, Keeney S, de Massy B, Jasin M. 2014 Mouse tetrad analysis provides insights into recombination mechanisms and hotspot evolutionary dynamics. Nat Genet. 46(10):1072–1080. 25151354 10.1038/ng.3068 PMC4207963

[msaa072-B21] Cole F, Keeney S, Jasin M. 2010 Comprehensive, fine-scale dissection of homologous recombination outcomes at a hot spot in mouse meiosis. Mol Cell. 39(5):700–710. 20832722 10.1016/j.molcel.2010.08.017 PMC3196603

[msaa072-B22] Coop G, Przeworski M. 2007 An evolutionary view of human recombination. Nat Rev Genet. 8(1):23–34. 17146469 10.1038/nrg1947

[msaa072-B23] Corcoran P, Gossmann TI, Barton HJ, Slate J, Zeng K, The Great Tit HapMap Consortium. 2017 Determinants of the efficacy of natural selection on coding and noncoding variability in two passerine species. Genome Biol Evol. 9(11):2987–3007. 29045655 10.1093/gbe/evx213 PMC5714183

[msaa072-B24] Costantini M, Cammarano R, Bernardi G. 2009 The evolution of isochore patterns in vertebrate genomes. BMC Genomics 10:146. 10.1186/1471-2164-10-146 PMC2678159 19344507

[msaa072-B25] Cox A, Ackert-Bicknell CL, Dumont BL, Ding Y, Bell JT, Brockmann GA, Wergedal JE, Bult C, Paigen B, Flint J, et al 2009 A new standard genetic map for the laboratory mouse. Genetics 182(4):1335–1344. 19535546 10.1534/genetics.109.105486 PMC2728870

[msaa072-B26] Dai Y, Holland P. 2019 The interaction of natural selection and GC skew may drive the fast evolution of a sand rat homeobox gene. Mol Biol Evol. 36(7):1473–1480. 30968125 10.1093/molbev/msz080 PMC6573468

[msaa072-B27] de la Fuente R, Manterola M, Viera A, Parra MT, Alsheimer M, Rufas JS, Page J. 2014 Chromatin organization and remodeling of interstitial telomeric sites during meiosis in the Mongolian gerbil (*Meriones unguiculatus*). Genetics 197(4):1137–1151. 24907260 10.1534/genetics.114.166421 PMC4125389

[msaa072-B28] Dreszer TR, Wall GD, Haussler D, Pollard KS. 2007 Biased clustered substitutions in the human genome: the footprints of male-driven biased gene conversion. Genome Res. 17(10):1420–1430. 17785536 10.1101/gr.6395807 PMC1987345

[msaa072-B29] Duret L, Arndt PF. 2008 The impact of recombination on nucleotide substitutions in the human genome. PLoS Genet. 4(5):e1000071. 18464896 10.1371/journal.pgen.1000071 PMC2346554

[msaa072-B30] Duret L, Galtier N. 2009 Biased gene conversion and the evolution of mammalian genomic landscapes. Annu Rev Genom Hum Genet. 10(1):285–311. 10.1146/annurev-genom-082908-150001 19630562

[msaa072-B31] Emms DM, Kelly S. 2015 OrthoFinder: solving fundamental biases in whole genome comparisons dramatically improves orthogroup inference accuracy. Genome Biol. 16:157. 10.1186/s13059-015-0721-2 PMC4531804 26243257

[msaa072-B32] Emms DM, Kelly S. 2019 OrthoFinder: phylogenetic orthology inference for comparative genomics. *Genome Biol.* 20(1):238. 10.1186/s13059-019-1832-y PMC6857279 31727128

[msaa072-B33] Eyre-Walker A. 1999 Evidence of selection on silent site base composition in mammals: potential implications for the evolution of isochores and junk DNA. Genetics 152(2):675–683. 10.1093/genetics/152.2.675 PMC1460637 10353909

[msaa072-B34] Ferguson-Smith MA, Trifonov V. 2007 Mammalian karyotype evolution. Nat Rev Genet. 8(12):950–962. 18007651 10.1038/nrg2199

[msaa072-B35] Figuet E, Ballenghien M, Romiguier J, Galtier N. 2015 Biased gene conversion and GC-content evolution in the coding sequences of reptiles and vertebrates. Genome Biol Evol. 7(1):240–250. 10.1093/gbe/evu277 PMC4316630 25527834

[msaa072-B36] Galtier N, Duret L. 2007 Adaptation or biased gene conversion? Extending the null hypothesis of molecular evolution. Trends Genet. 23(6):273–277. 17418442 10.1016/j.tig.2007.03.011

[msaa072-B37] Galtier N, Duret L, Glémin S, Ranwez V. 2009 GC-biased gene conversion promotes the fixation of deleterious amino acid changes in primates. Trends Genet. 25(1):1–5. 19027980 10.1016/j.tig.2008.10.011

[msaa072-B38] Galtier N, Piganeau G, Mouchiroud D, Duret L. 2001 GC-content evolution in mammalian genomes: the biased gene conversion hypothesis. Genetics 159(2):907–911. 11693127 10.1093/genetics/159.2.907 PMC1461818

[msaa072-B39] Galtier N, Roux C, Rousselle M, Romiguier J, Figuet E, Glémin S, Bierne N, Duret L. 2018 Codon usage bias in animals: disentangling the effects of natural selection, effective population size, and GC-biased gene conversion. Mol Biol Evol. 35(5):1092–1103. 29390090 10.1093/molbev/msy015

[msaa072-B40] Glémin S, Arndt PF, Messer PW, Petrov D, Galtier N, Duret L. 2015 Quantification of GC-biased gene conversion in the human genome. Genome Res. 25(8):1215–1228. 25995268 10.1101/gr.185488.114 PMC4510005

[msaa072-B41] Grabherr MG, Haas BJ, Yassour M, Levin JZ, Thompson DA, Amit I, Adiconis X, Fan L, Raychowdhury R, Zeng Q, et al 2011 Full-length transcriptome assembly from RNA-Seq data without a reference genome. Nat Biotechnol. 29(7):644–652. 21572440 10.1038/nbt.1883 PMC3571712

[msaa072-B42] Guéguen L, Gaillard S, Boussau B, Gouy M, Groussin M, Rochette NC, Bigot T, Fournier D, Pouyet F, Cahais V, et al 2013 Bio++: efficient extensible libraries and tools for computational molecular evolution. Mol Biol Evol. 30(8):1745–1750. 23699471 10.1093/molbev/mst097

[msaa072-B43] Halldorsson BV, Hardarson MT, Kehr B, Styrkarsdottir U, Gylfason A, Thorleifsson G, Zink F, Jonasdottir A, Jonasdottir A, Sulem P, et al 2016 The rate of meiotic gene conversion varies by sex and age. Nat Genet. 48(11):1377–1384. 27643539 10.1038/ng.3669 PMC5083143

[msaa072-B44] Hargreaves AD, Zhou L, Christensen J, Marlétaz F, Liu S, Li F, Jansen PG, Spiga E, Hansen MT, Pedersen SVH, et al 2017 Genome sequence of a diabetes-prone rodent reveals a mutation hotspot around the ParaHox gene cluster. Proc Natl Acad Sci USA. 114(29):7677–7682. 28674003 10.1073/pnas.1702930114 PMC5530673

[msaa072-B45] Jensen-Seaman MI, Furey TS, Payseur BA, Lu Y, Roskin KM, Chen C-F, Thomas MA, Haussler D, Jacob HJ. 2004 Comparative recombination rates in the rat, mouse, and human genomes. Genome Res. 14(4):528–538. 15059993 10.1101/gr.1970304 PMC383296

[msaa072-B46] Joshi NA, Fass JN. 2011 Sickle: A sliding-window, adaptive, quality-based trimming tool for FastQ files (Version 1.33). Available from: https://github.com/najoshi/sickle. Accessed March 30, 2020.

[msaa072-B47] Katzman S, Capra JA, Haussler D, Pollard KS. 2011 Ongoing GC-biased evolution is widespread in the human genome and enriched near recombination hot spots. Genome Biol Evol. 3:614–626. 21697099 10.1093/gbe/evr058 PMC3157837

[msaa072-B48] Kostka D, Hubisz MJ, Siepel A, Pollard KS. 2012 The role of GC-biased gene conversion in shaping the fastest evolving regions of the human genome. Mol Biol Evol. 29(3):1047–1057. 22075116 10.1093/molbev/msr279 PMC3278478

[msaa072-B49] Lamb BC. 1984 The properties of meiotic gene conversion important in its effects on evolution. Heredity 53(1):113–138. 6436195 10.1038/hdy.1984.68

[msaa072-B50] Latrille T, Duret L, Lartillot N. 2017 The Red Queen model of recombination hot-spot evolution: a theoretical investigation. Philos Trans R Soc B. 372(1736):20160463. 10.1098/rstb.2016.0463 PMC5698625 29109226

[msaa072-B51] Li R, Bitoun E, Altemose N, Davies RW, Davies B, Myers SR. 2019 A high-resolution map of non-crossover events reveals impacts of genetic diversity on mammalian meiotic recombination. Nat Commun. 10(1):3900. 10.1038/s41467-019-11675-y PMC6715734 31467277

[msaa072-B52] Long H, Sung W, Kucukyildirim S, Williams E, Miller SF, Guo W, Patterson C, Gregory C, Strauss C, Stone C, et al 2018 Evolutionary determinants of genome-wide nucleotide composition. Nat Ecol Evol. 2(2):237–240. 29292397 10.1038/s41559-017-0425-y PMC6855595

[msaa072-B53] Lynch M. 2007. The origins of genome architecture. Sunderland (MA): Sinauer Associates Incorporated.

[msaa072-B54] Marais G, Mouchiroud D, Duret L. 2001 Does recombination improve selection on codon usage? Lessons from nematode and fly complete genomes. Proc Natl Acad Sci USA. 98(10):5688–5692. 11320215 10.1073/pnas.091427698 PMC33274

[msaa072-B55] Mugal CF, Arndt PF, Ellegren H. 2013 Twisted signatures of GC-biased gene conversion embedded in an evolutionary stable karyotype. Mol Biol Evol. 30(7):1700–1712. 23564940 10.1093/molbev/mst067 PMC3684855

[msaa072-B56] Munch K, Mailund T, Dutheil JY, Schierup MH. 2014 A fine-scale recombination map of the human–chimpanzee ancestor reveals faster change in humans than in chimpanzees and a strong impact of GC-biased gene conversion. Genome Res. 24(3):467–474. 24190946 10.1101/gr.158469.113 PMC3941111

[msaa072-B57] Myers S, Bowden R, Tumian A, Bontrop RE, Freeman C, MacFie TS, McVean G, Donnelly P. 2010 Drive against hotspot motifs in primates implicates the PRDM9 gene in meiotic recombination. Science 327(5967):876–879. 20044541 10.1126/science.1182363 PMC3828505

[msaa072-B58] Nabholz B, Künstner A, Wang R, Jarvis ED, Ellegren H. 2011 Dynamic evolution of base composition: causes and consequences in avian phylogenomics. Mol Biol Evol. 28(8):2197–2210. 21393604 10.1093/molbev/msr047 PMC3144382

[msaa072-B59] Necşulea A, Popa A, Cooper DN, Stenson PD, Mouchiroud D, Gautier C, Duret L. 2011 Meiotic recombination favors the spreading of deleterious mutations in human populations. Hum Mutat. 32(2):198–206. 21120948 10.1002/humu.21407

[msaa072-B60] Odenthal-Hesse L, Berg IL, Veselis A, Jeffreys AJ, May CA. 2014 Transmission distortion affecting human noncrossover but not crossover recombination: a hidden source of meiotic drive. PLoS Genet. 10(2):e1004106. 24516398 10.1371/journal.pgen.1004106 PMC3916235

[msaa072-B61] Pessia E, Popa A, Mousset S, Rezvoy C, Duret L, Marais G. 2012 Evidence for widespread GC-biased gene conversion in eukaryotes. Genome Biol Evol. 4(7):675–682. 22628461 10.1093/gbe/evs052 PMC5635611

[msaa072-B62] Pracana R, Hargreaves A. 2019 Analyses of GC evolution in gerbil genomes. 2019. Oxford University Research Archive. 10.5287/bodleian:mzQBXzGJ8. Accessed March 30, 2020.

[msaa072-B63] Pratto F, Brick K, Khil P, Smagulova F, Petukhova GV, Camerini-Otero RD. 2014 Recombination initiation maps of individual human genomes. Science 346(6211):1256442. 25395542 10.1126/science.1256442 PMC5588152

[msaa072-B64] Ranwez V, Douzery EJP, Cambon C, Chantret N, Delsuc F. 2018 MACSE v2: toolkit for the alignment of coding sequences accounting for frameshifts and stop codons. Mol Biol Evol. 35(10):2582–2584. 30165589 10.1093/molbev/msy159 PMC6188553

[msaa072-B65] Ratnakumar A, Mousset S, Glémin S, Berglund J, Galtier N, Duret L, Webster MT. 2010 Detecting positive selection within genomes: the problem of biased gene conversion. Philos Trans R Soc B. 365(1552):2571–2580. 10.1098/rstb.2010.0007 PMC2935097 20643747

[msaa072-B66] Romiguier J, Figuet E, Galtier N, Douzery EJP, Boussau B, Dutheil JY, Ranwez V. 2012 Fast and robust characterization of time-heterogeneous sequence evolutionary processes using substitution mapping. PLoS One 7(3):e33852. 22479459 10.1371/journal.pone.0033852 PMC3313935

[msaa072-B67] Romiguier J, Ranwez V, Douzery EJP, Galtier N. 2010 Contrasting GC-content dynamics across 33 mammalian genomes: relationship with life-history traits and chromosome sizes. Genome Res. 20(8):1001–1009. 20530252 10.1101/gr.104372.109 PMC2909565

[msaa072-B68] Rousselle M, Laverré A, Figuet E, Nabholz B, Galtier N. 2019 Influence of recombination and GC-biased gene conversion on the adaptive and nonadaptive substitution rate in mammals versus birds. Mol Biol Evol. 36(3):458–471. 30590692 10.1093/molbev/msy243 PMC6389324

[msaa072-B69] Saccone S, Federico C, Bernardi G. 2002 Localization of the gene-richest and the gene-poorest isochores in the interphase nuclei of mammals and birds. Gene 300(1–2):169–178. 12468098 10.1016/s0378-1119(02)01038-7

[msaa072-B70] Singhal S, Leffler EM, Sannareddy K, Turner I, Venn O, Hooper DM, Strand AI, Li Q, Raney B, Balakrishnan CN, et al 2015 Stable recombination hotspots in birds. Science 350(6263):928–932. 26586757 10.1126/science.aad0843 PMC4864528

[msaa072-B71] Smagulova F, Brick K, Pu Y, Camerini-Otero RD, Petukhova GV. 2016 The evolutionary turnover of recombination hot spots contributes to speciation in mice. Genes Dev. 30(3):266–280. 26833728 10.1101/gad.270009.115 PMC4743057

[msaa072-B72] Smeds L, Mugal CF, Qvarnström A, Ellegren H. 2016 High-resolution mapping of crossover and non-crossover recombination events by whole-genome re-sequencing of an avian pedigree. PLoS Genet. 12(5):e1006044. 27219623 10.1371/journal.pgen.1006044 PMC4878770

[msaa072-B73] Smith TCA, Arndt PF, Eyre-Walker A. 2018 Large scale variation in the rate of germ-line de novo mutation, base composition, divergence and diversity in humans. PLoS Genet. 14(3):e1007254. 29590096 10.1371/journal.pgen.1007254 PMC5891062

[msaa072-B74] Spencer CCA, Deloukas P, Hunt S, Mullikin J, Myers SR, Silverman B, Donnelly P, Bentley D, McVean G. 2006 The influence of recombination on human genetic diversity. PLoS Genet. 2(9):e148. 17044736 10.1371/journal.pgen.0020148 PMC1575889

[msaa072-B75] Stapley J, Feulner PGD, Johnston SE, Santure AW, Smadja CM. 2017 Variation in recombination frequency and distribution across eukaryotes: patterns and processes. Philos Trans R Soc B. 372(1736):20160455. 10.1098/rstb.2016.0455 PMC5698618 29109219

[msaa072-B76] Steppan S, Adkins R, Anderson J. 2004 Phylogeny and divergence-date estimates of rapid radiations in muroid rodents based on multiple nuclear genes. Syst Biol. 53(4):533–553. 15371245 10.1080/10635150490468701

[msaa072-B77] Vinogradov AE. 2003 DNA helix: the importance of being GC-rich. Nucleic Acids Res. 31(7):1838–1844. 12654999 10.1093/nar/gkg296 PMC152811

[msaa072-B78] Weber CC, Boussau B, Romiguier J, Jarvis ED, Ellegren H. 2014 Evidence for GC-biased gene conversion as a driver of between-lineage differences in avian base composition. Genome Biol. 15(12):549. 10.1186/s13059-014-0549-1 PMC4290106 25496599

[msaa072-B79] Webster MT, Smith N. 2004 Fixation biases affecting human SNPs. Trends Genet. 20(3):122–126. 15049304 10.1016/j.tig.2004.01.005

[msaa072-B80] Williams AL, Genovese G, Dyer T, Altemose N, Truax K, Jun G, Patterson N, Myers SR, Curran JE, Duggirala R, et al 2015 Non-crossover gene conversions show strong GC bias and unexpected clustering in humans. Elife 4:e04637. 10.7554/eLife.04637 PMC4404656 25806687

[msaa072-B81] Yang Z, Nielsen R. 1998 Synonymous and nonsynonymous rate variation in nuclear genes of mammals. J Mol Evol. 46(4):409–418. 9541535 10.1007/pl00006320

[msaa072-B82] Zamudio N, Barau J, Teissandier A, Walter M, Borsos M, Servant N, Bourc'his D. 2015 DNA methylation restrains transposons from adopting a chromatin signature permissive for meiotic recombination. Genes Dev. 29(12):1256–1270. 26109049 10.1101/gad.257840.114 PMC4495397

