# Supplementary material for: Runaway GC Evolution in Gerbil Genomes
Source: Mol Biol Evol. 2020 Apr 24;37(8):2197–210. doi: 10.1093/molbev/msaa072 (PMC7403616; doi:10.1093/molbev/msaa072)
Supplement: msaa072_Supplementary_Figures_Tables [file msaa072_supplementary_figures_tables.pdf]

Runaway GC evolution in gerbil genomes

Supplementary Figures and Supplementary Tables

Rodrigo Pracana [1]
Adam D. Hargreaves [1]
John F. Mulley [2]
Peter W. H. Holland [1]

[1] Department of Zoology, 11a Mansfield Road, University of Oxford, Oxford OX1 3SZ, United Kingdom
[2] School of Natural Sciences, Bangor University, Brambell Building, Deiniol Road, Bangor, Gwynedd LL57 2UW, United Kingdom

Supplementary Figures

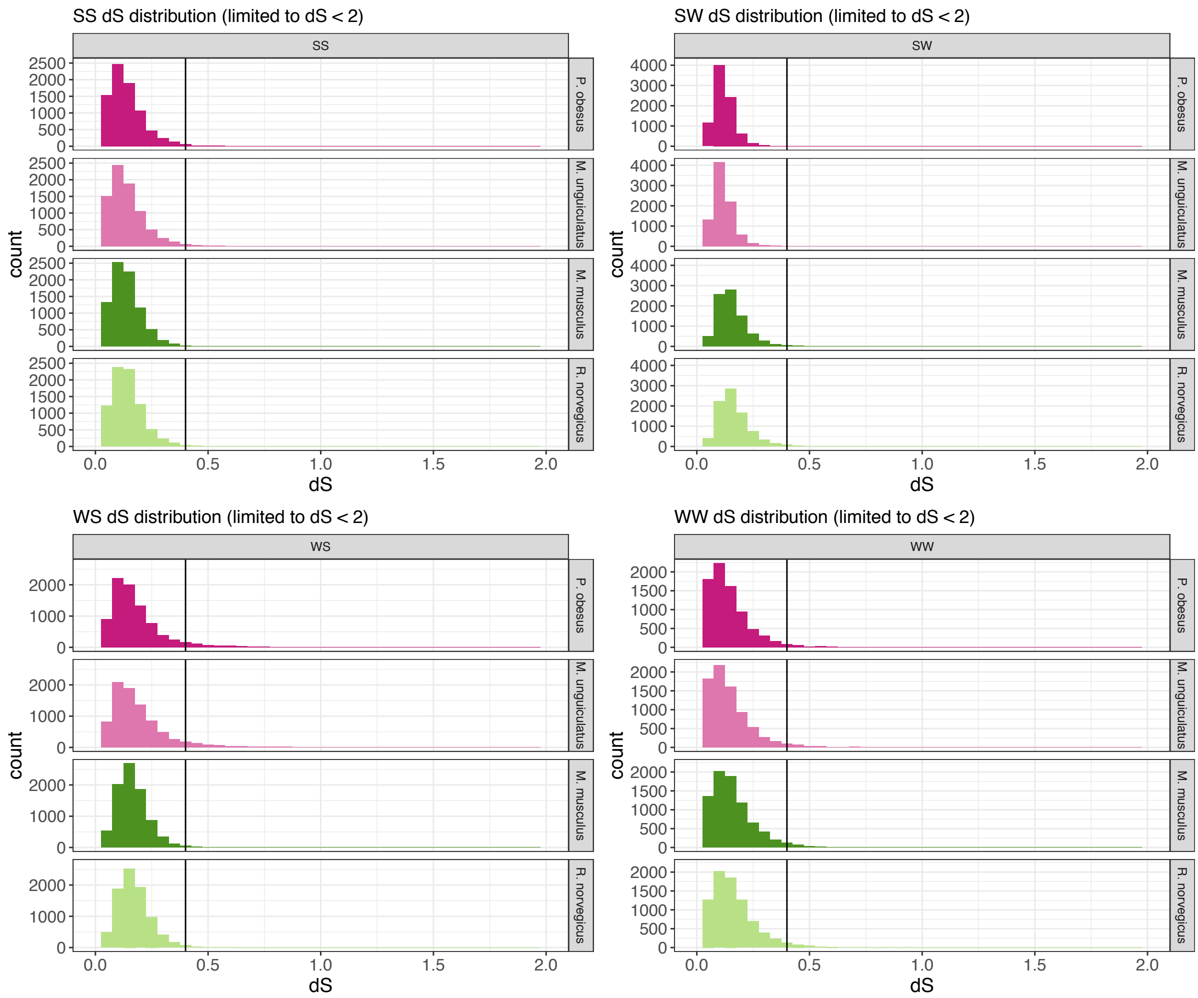

Supplementary Figure 1

Distribution of the rate of synonymous substitutions across 8,809 orthologous groups for each mutational category: strong to strong (SS), strong to weak (SW), weak to strong (WS) and weak to weak (WW). Only values under 2 are shown.

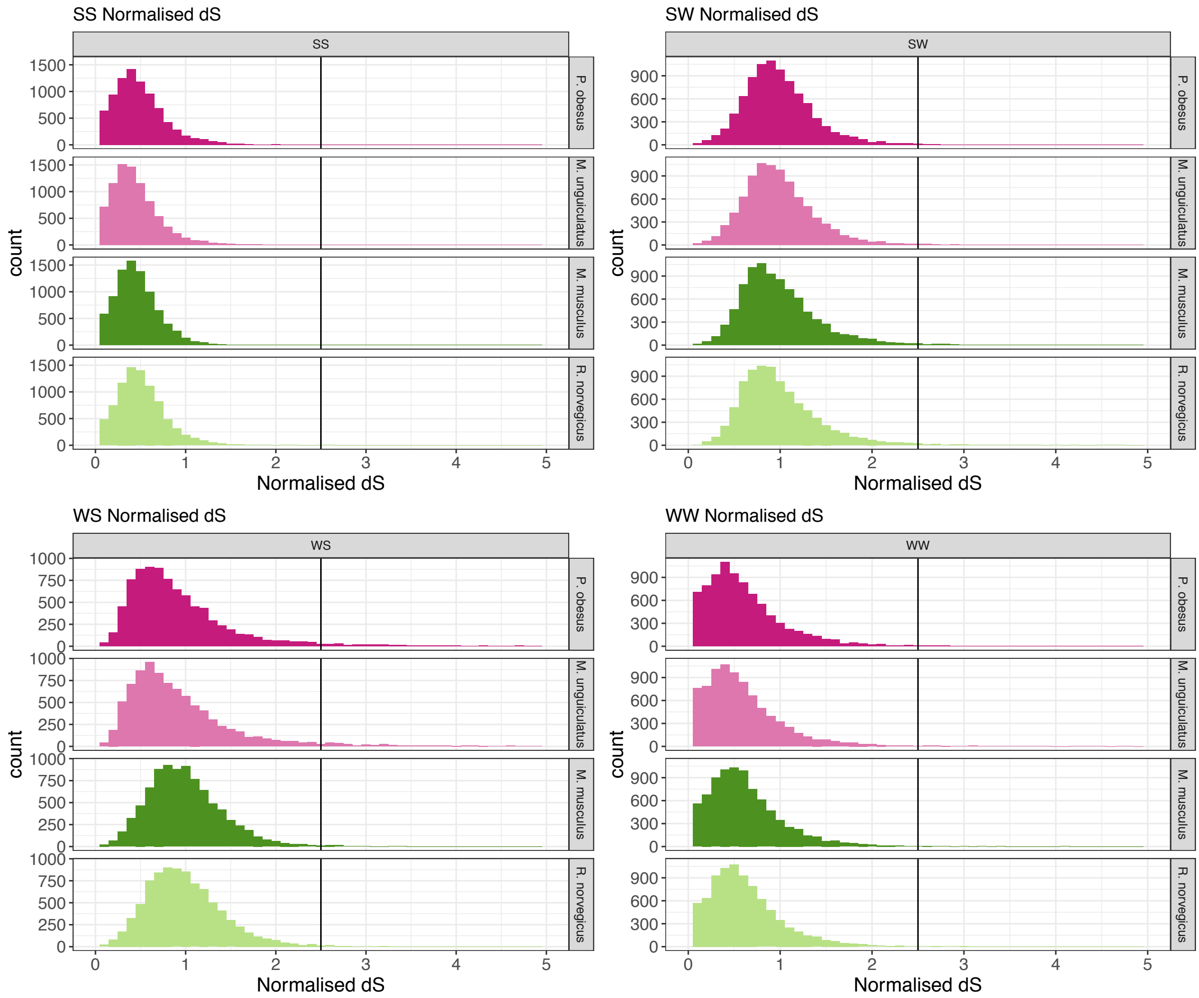

Supplementary Figure 2

Distribution of the normalised rate of synonymous substitutions across 8,809 orthologous groups for each mutational category: strong to strong (SS), strong to weak (SW), weak to strong (WS) and weak to weak (WW). For each category and each species, we didvided the raw dS values by the respective average value. The vertical grey line represents the dS threshold chosen to define outlier genes (2.5 times the average of the respective group). Only values under 5 are shown.

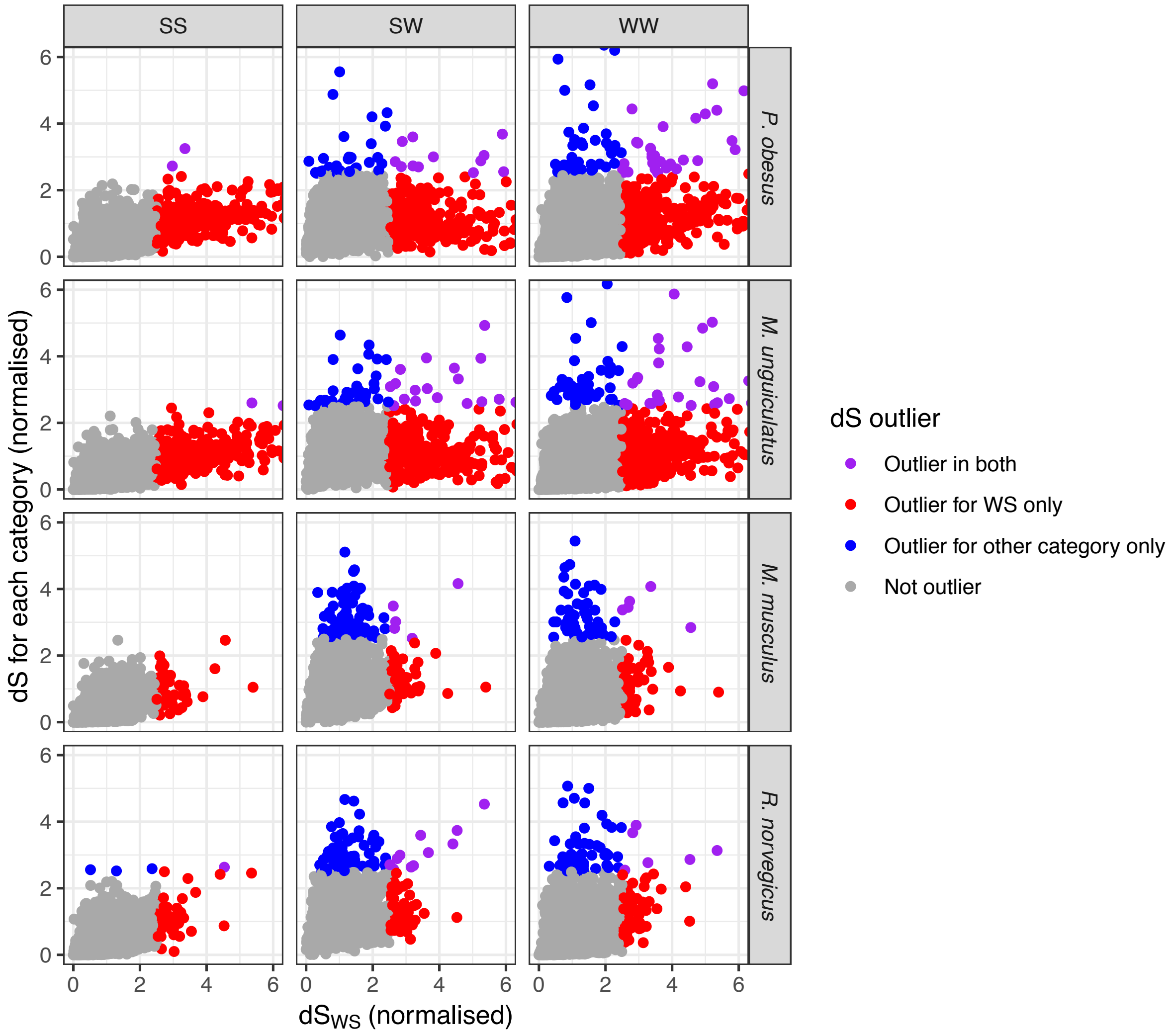

Supplementary Figure 3

Comparison between the rate of weak-to-strong synonymous substitution (dS_{WS}) and dS for the other three categories, strong-to-strong (SS), strong-to-weak (SW) and weak-to-weak (WW) in the two gerbil and the two murine species. Coloured points represent genes above the dS threshold chosen to define outliers (dS larger than 2.5 times the average rate for the respective species and mutational category). Only those genes with dS values below 6 are shown.

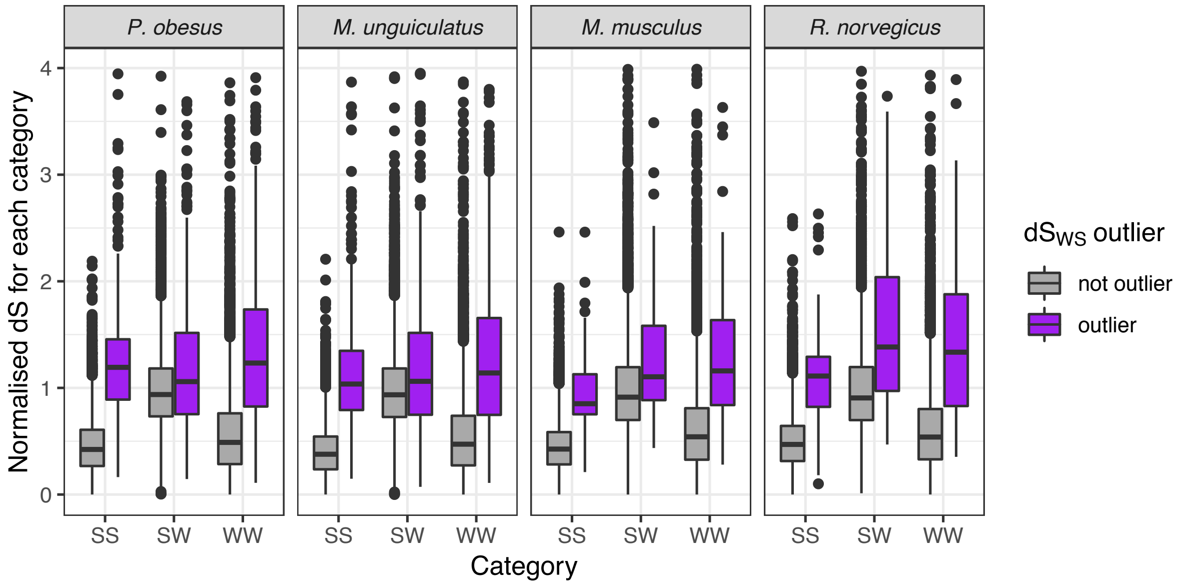

Supplementary Figure 4

Difference in dS rate for three mutational categories (SS, SW and WW) between genes that are classed as outliers in dS_{WS} and those that are not. The difference is statistically significant for all categories (Supplementary Table 2).

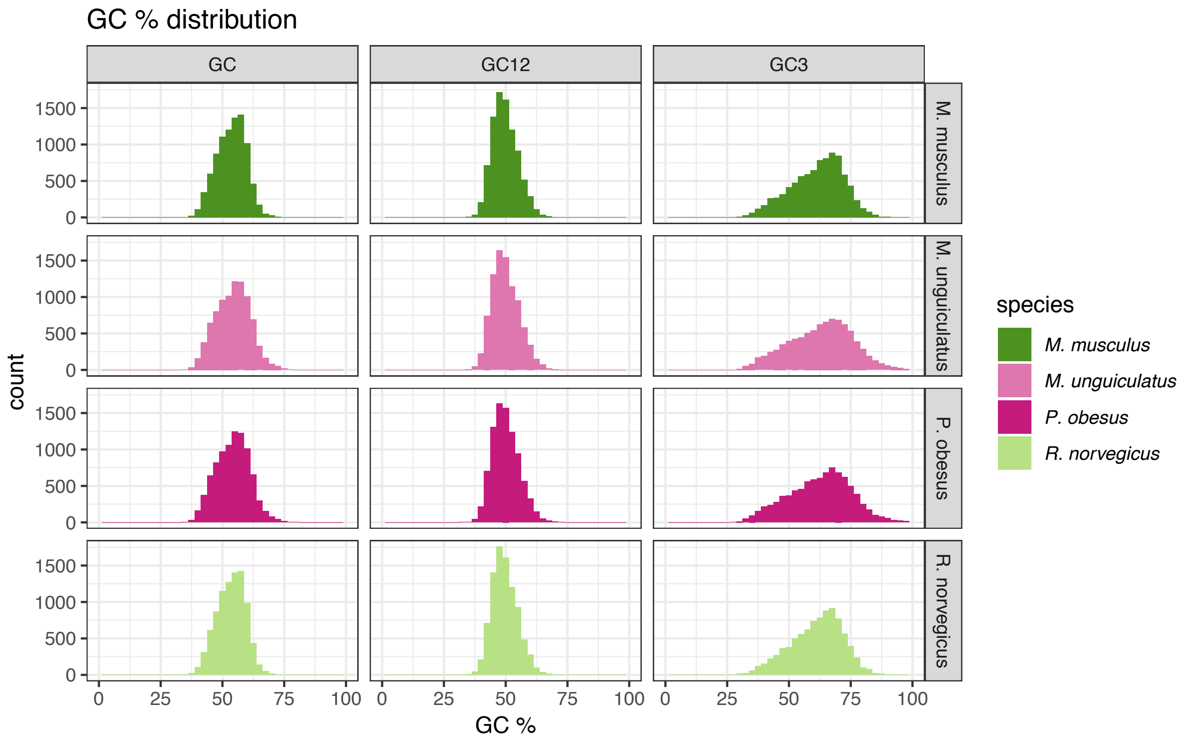

Supplementary Figure 5

GC content in all codon positions (GC), in first and second codon positions (GC12) and in the third codon position (GC3) per species across 8,809 groups of orthologous genes (we did not consider genes for which the size of the coding sequence annotation was not a multiple of three: 12 genes in *M. musculus*, 5 in *M. unguiculatus*, 2 in *R. norvegicus* and none in *P. obesus*).

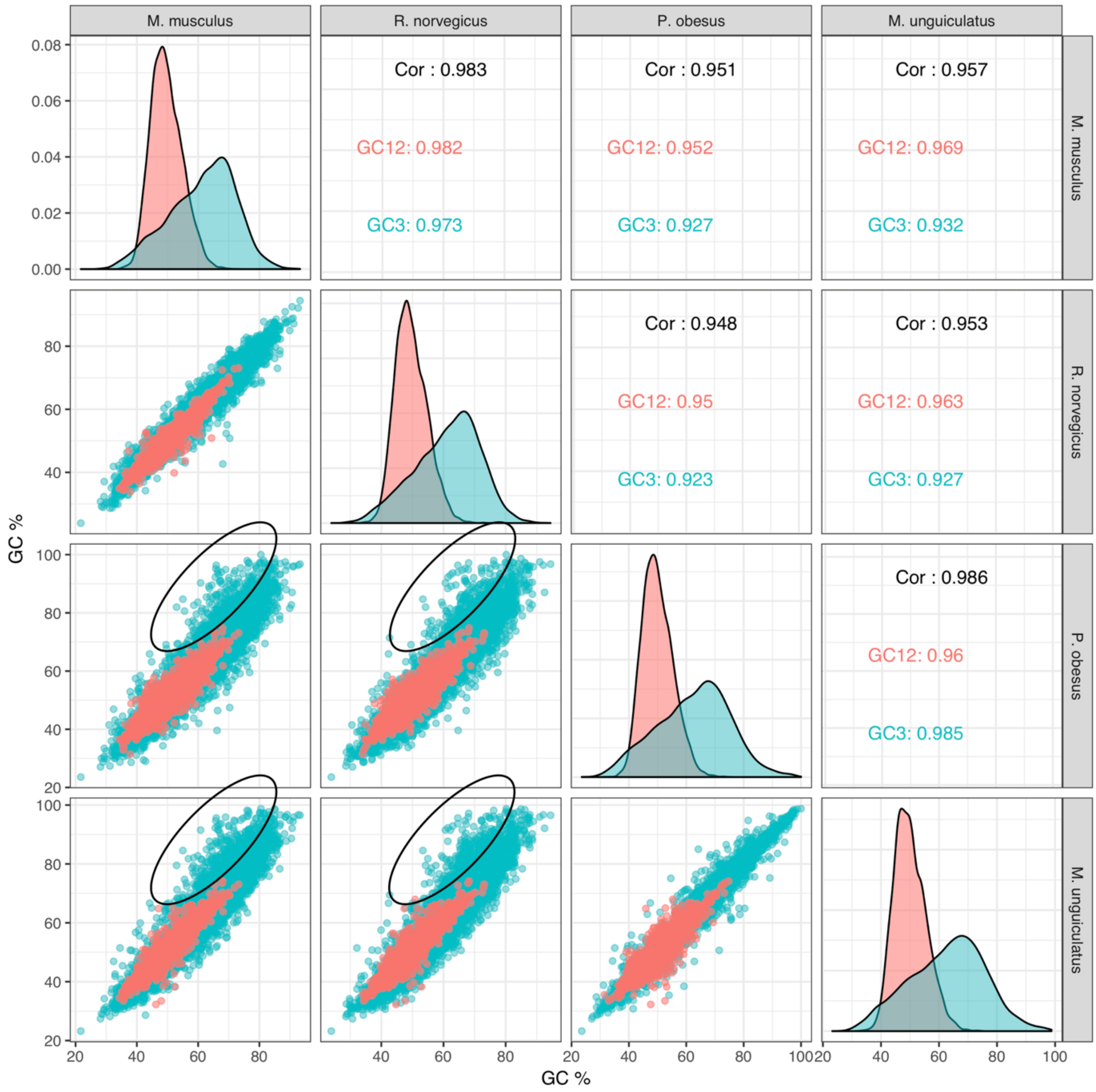

Supplementary Figure 6

Lower triangle: pairwise comparisons between the GC content of the different species with GC12 in red and GC3 in teal and a black line encircling the asymmetry in the distribution where the gerbil species have higher GC3 values than the murine species. Diagonal: density distribution of GC12 and GC3 in each species. Upper triangle: pairwise correlation values in GC12 and GC3 between each species. We did not consider genes for which the size of the coding sequence annotation was not a multiple of three: 12 genes in *M. musculus*, 5 in *M. unguiculatus*, 2 in *R. norvegicus* and none in *P. obesus*.

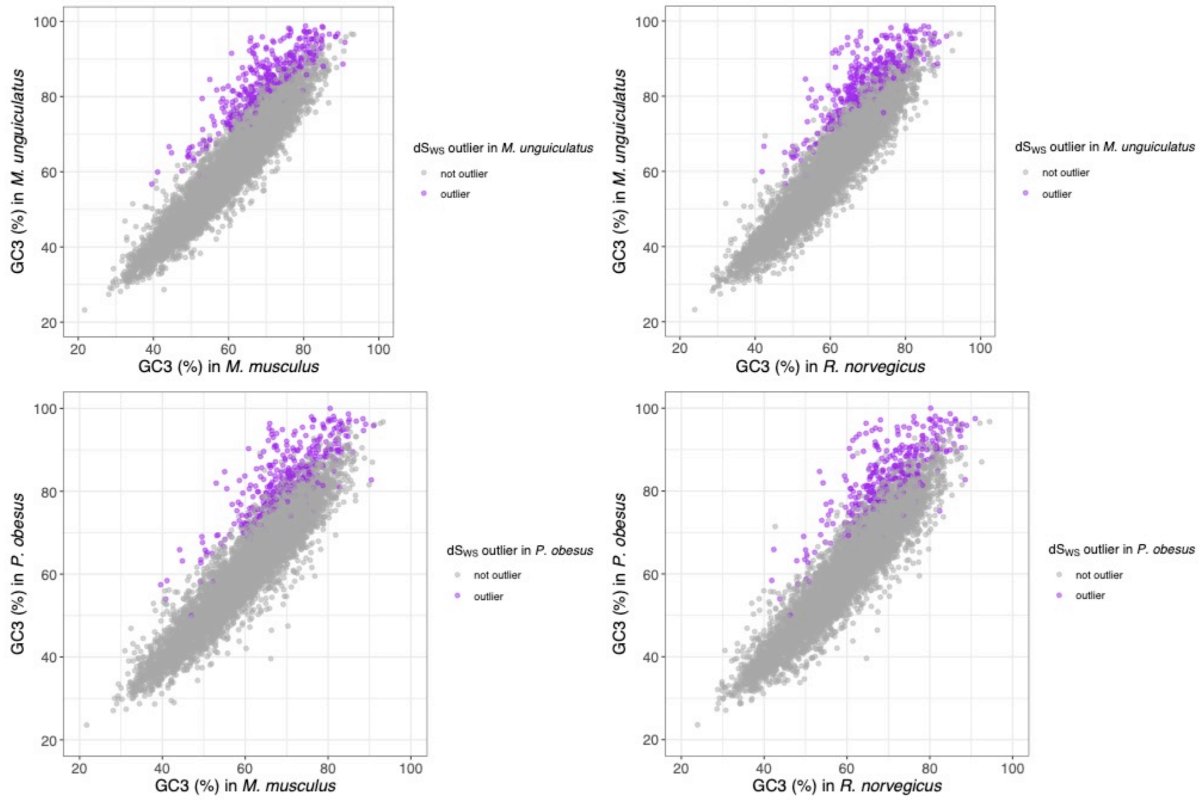

Supplementary Figure 7

Pairwise comparisons between the GC content at the third codon position (GC3) of the two murine species and the two gerbil species in 8,809 orthologous groups, with dS_{WS} outliers coloured purple. For each comparison, we removed any gene with no GC3 measurement for one of the two species.

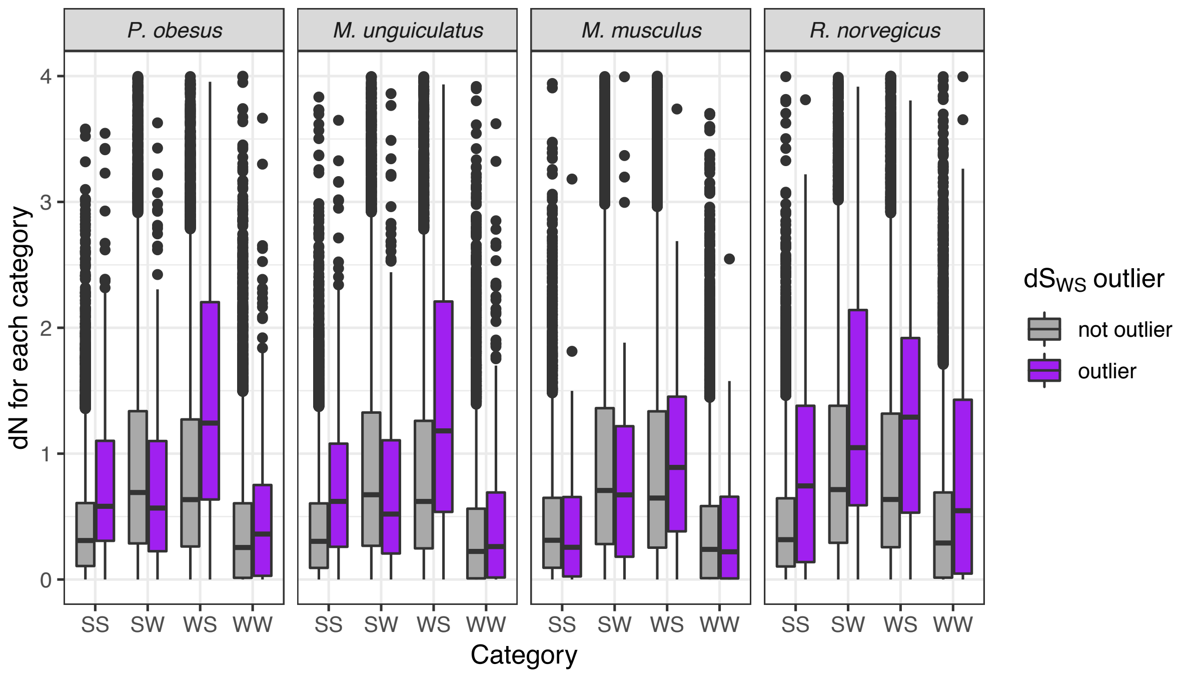

Supplementary Figure 8

dN distribution for the four mutational categories (strong-to-strong: SS; strong-to-weak: SW; weak-to-strong; WS; weak-to-weak: WW) between genes that are classed as outliers in dS_{WS} and those that are not. The difference is statistically significant for some of the categories, as listed in Supplementary Table 3.

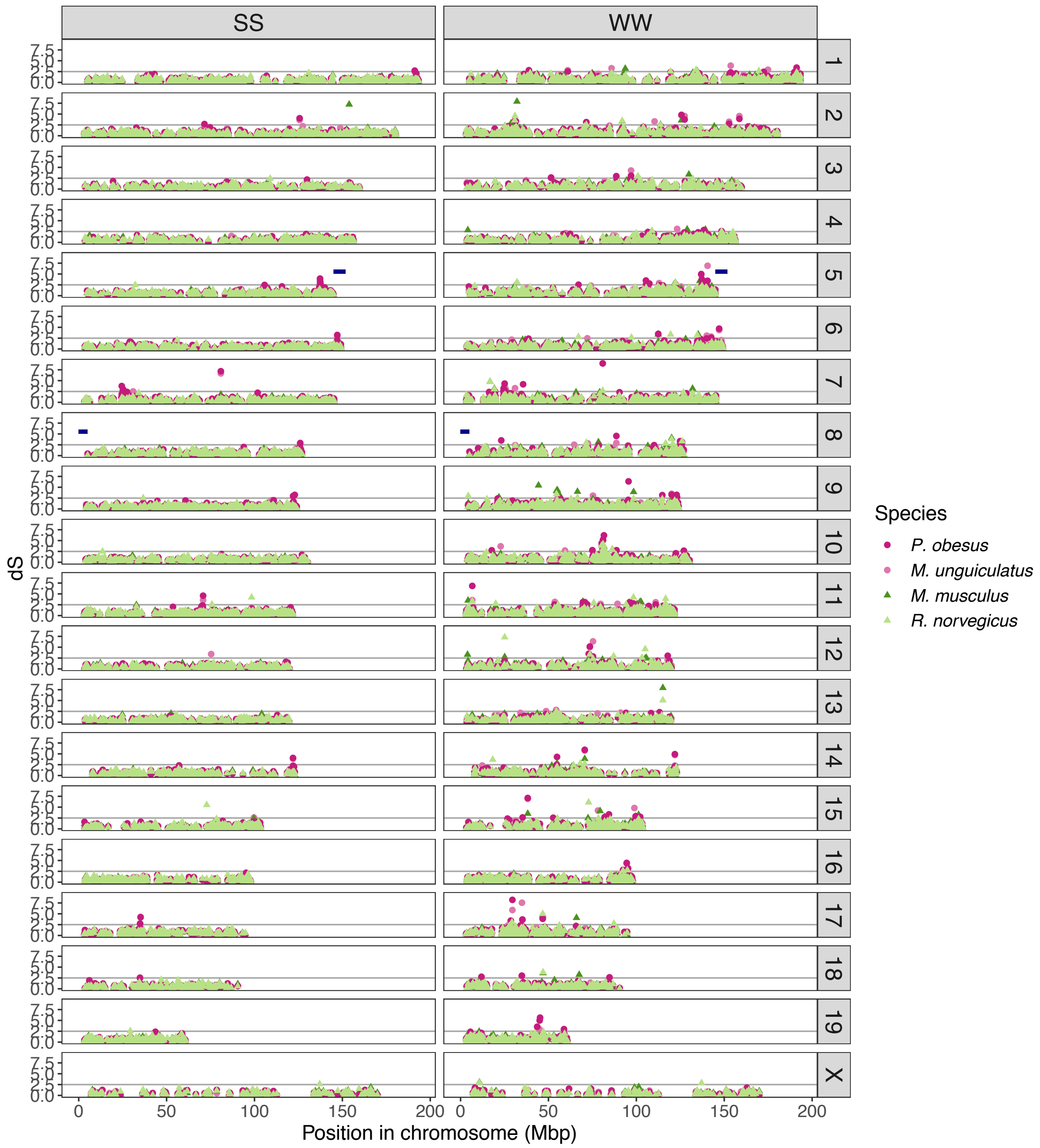

Supplementary Figure 9

Normalised rate of synonymous substitution (dS) for two mutational categories, strong to strong (SS) and weak to weak (WW), for 8,809 genes. The genes are mapped by row to the chromosomes of the *M. musculus* reference genome assembly. The horizontal grey line represents the dS threshold chosen to define outlier genes (dS larger than 2.5 times the average for the respective species and mutational category). The dark blue horizontal bars represent the location of the previously known high GC region.

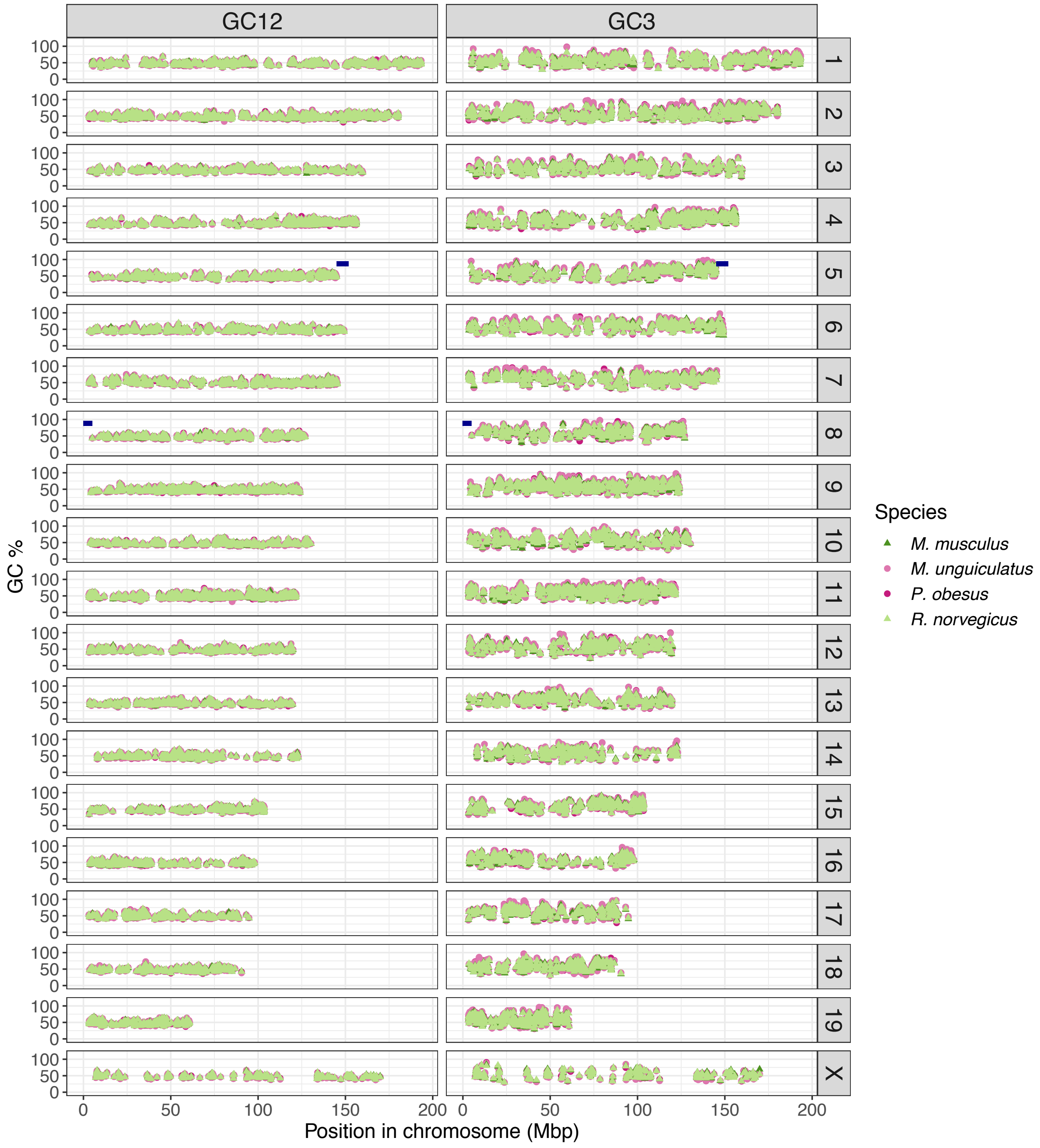

Supplementary Figure 10

GC content in the first and second codon positions (GC12) and in the third codon position (GC3) per species across 8,809 genes, mapped to the mouse chromosome assembly. The dark blue horizontal bars represent the location of the previously known high GC region.

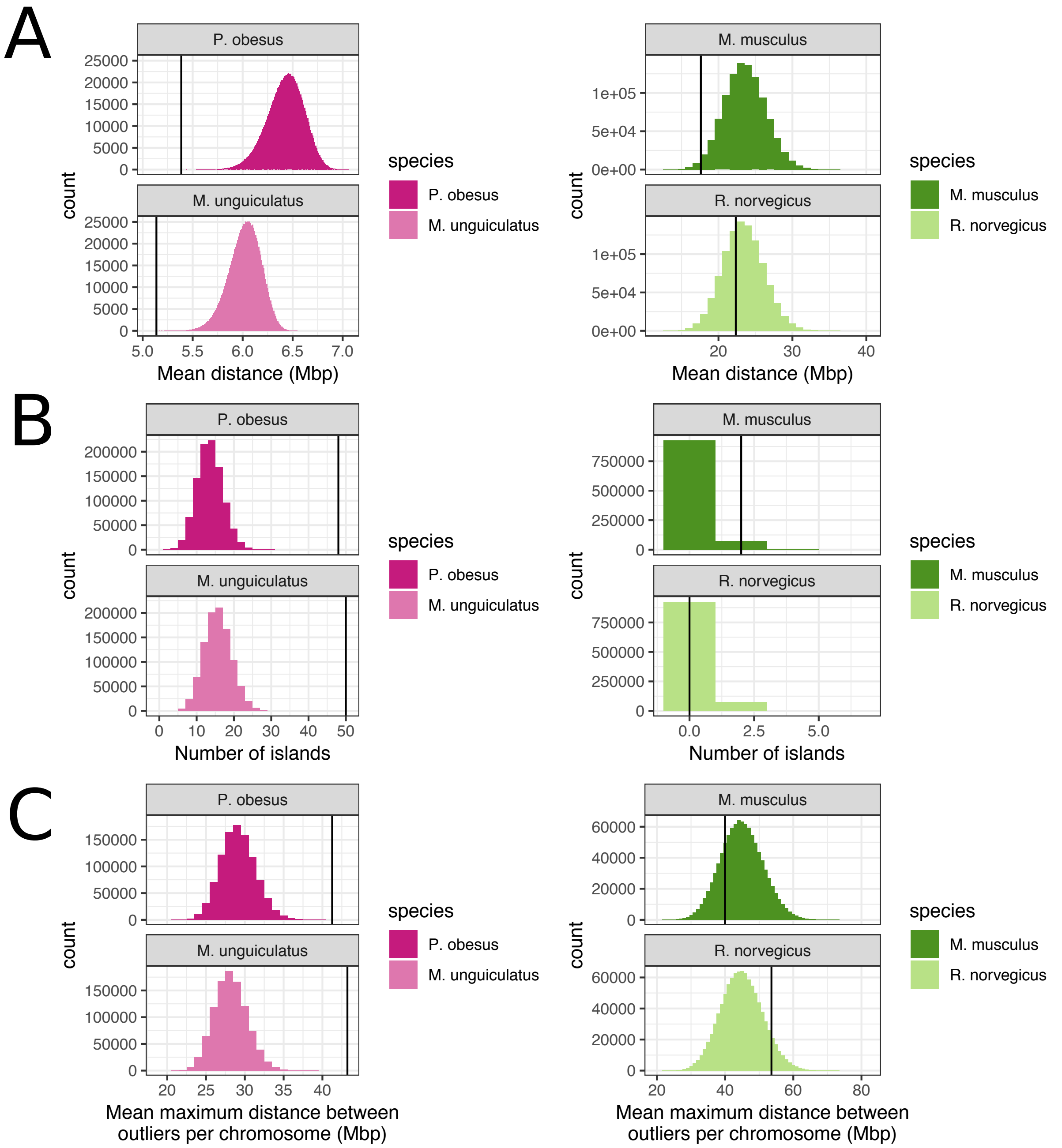

Supplementary Figure 11

Null distributions of the distances between dS_{WS} outliers, as inferred from randomising which genes are outliers in permutations (1,000,000 permutations per species, the number of outliers per species is given in Supplementary Table 1). Vertical lines represent the observed values. (A) Average distance between outliers for each permutation; (B) number of "islands", defined as runs of two or more neighbouring outlier genes; (C) maximum distance between outliers per chromosome, averaged for each permutation. Note the different scales for the gerbil and the murine species, resulting from the much higher number of outliers in the gerbils.

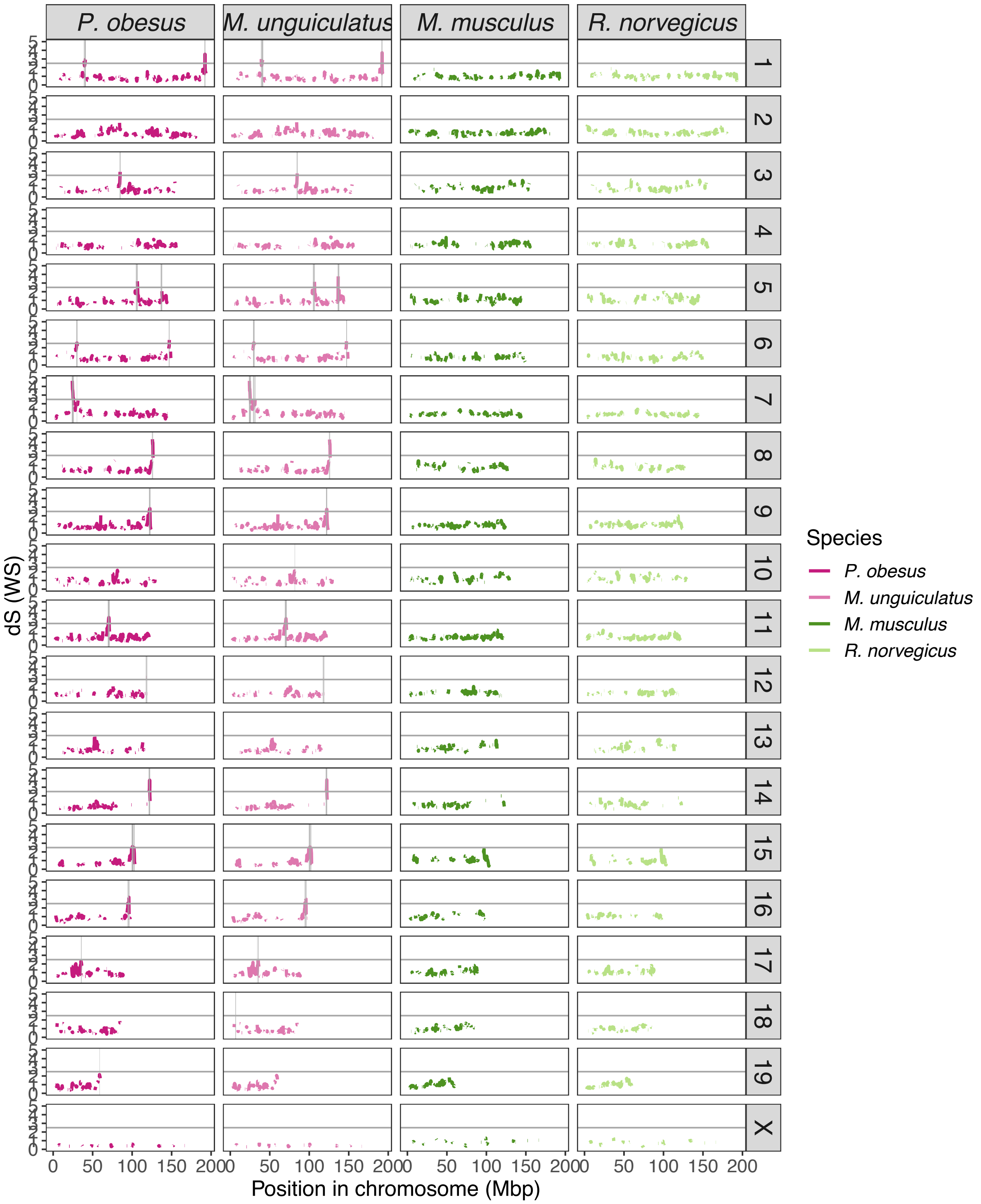

Supplementary Figure 12

Average normalised weak-to-strong dS (dS_{ws}) in sliding windows of 1 Mbp with a step of 0.25 Mbp, showing only windows with more than 3 genes, and with outlying regions marked with a vertical grey bar. The genes are mapped by row to the chromosomes of the *M. musculus* reference genome assembly. The horizontal grey line represents the dS threshold chosen to define outlier genes (dS_{ws} larger than 2.5 times its average for the respective species).

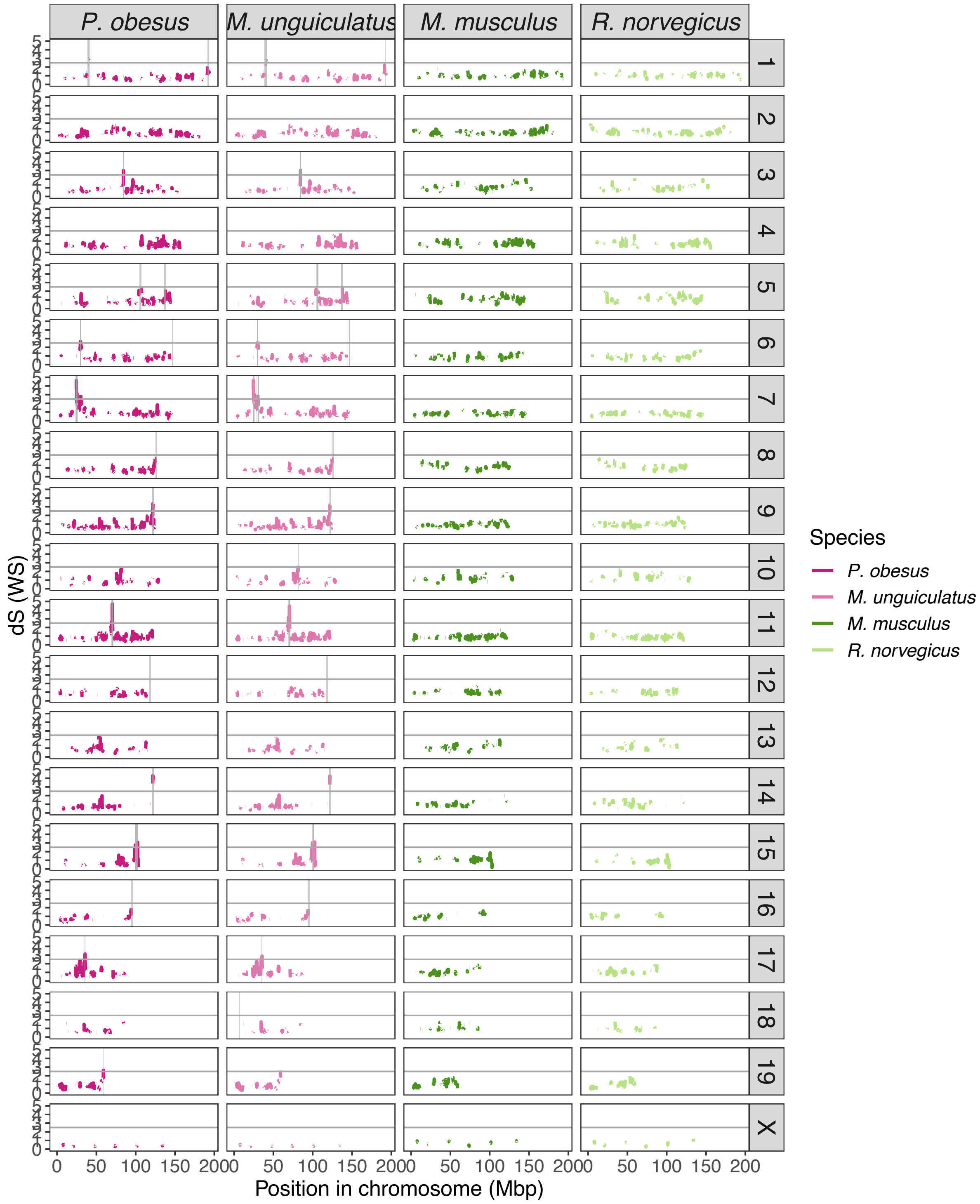

Supplementary Figure 13

Average normalised weak-to-strong dS (dS_{ws}) in sliding windows of 0.5 Mbp with a step of 0.1 Mbp, showing only windows with more than 3 genes, and with outlying regions marked with a vertical grey bar. The genes are mapped by row to the chromosomes of the *M. musculus* reference genome assembly. The horizontal grey line represents the dS threshold chosen to define outlier genes (dS_{ws} larger than 2.5 times its average for the respective species).

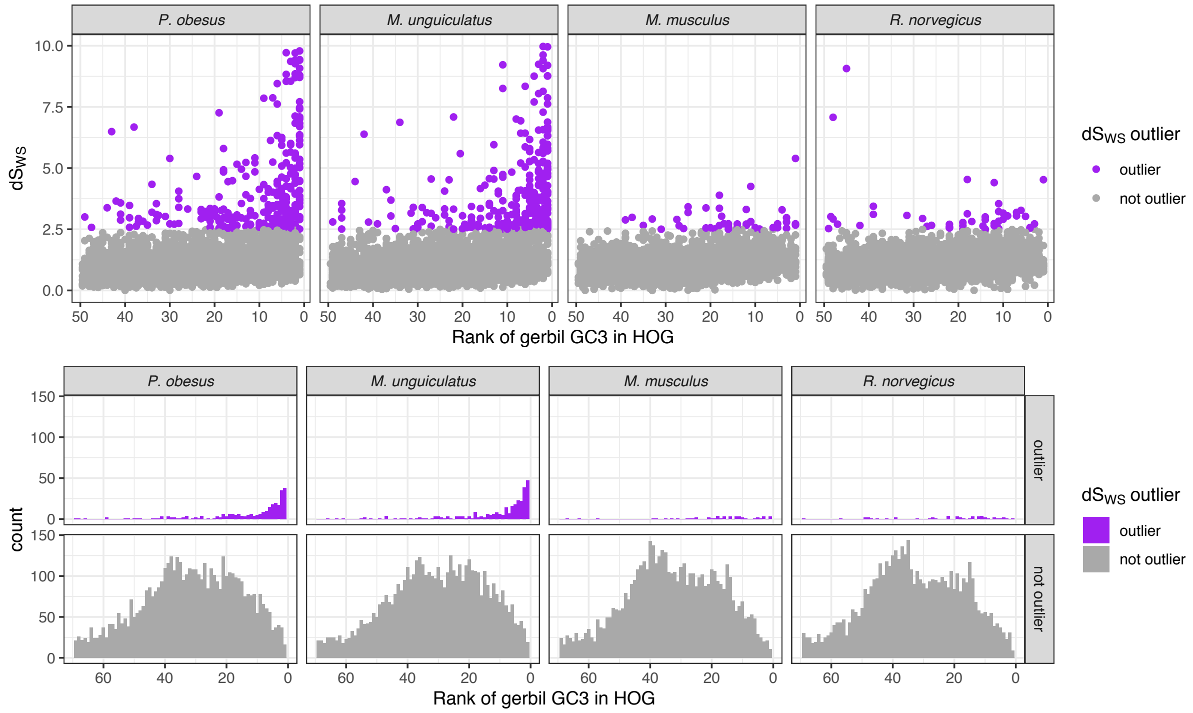

Supplementary Figure 14

Rank of murid genes in their assigned Hierarchical Orthologous Group (HOG).

[top] Genes with a high GC3 rank tend to have high dS_{WS} (one-sided Wilcoxon rank sum test comparing dS_{WS} outliers with the GC3 rank for each species; $P = 1.61 \times 10^{-93}$ for *P. obesus*; $P = 1.29 \times 10^{-100}$ for *M. unguiculatus*; $P = 7.53 \times 10^{-15}$ for *M. musculus*; $P = 6.90 \times 10^{-7}$ for *R. norvegicus*).

[bottom] dS_{WS} outliers are enriched for genes with a high rank of GC3 (Supplementary Table 8).

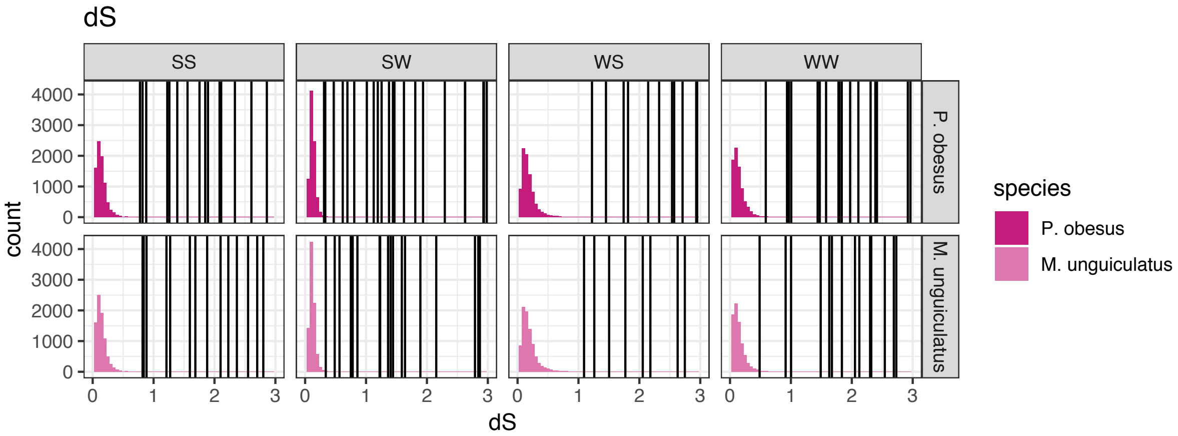

Supplementary Figure 15

Comparison between the rates of synonymous substitution (dS) for each mutational category (SS, SW, WS and WW) between the genes in the previously known region (black vertical lines) and genome-wide distribution estimated from the set of 8,909 groups of orthologous genes (coloured distributions). The values shown are not normalised, and limited to the range from 0 to 3.

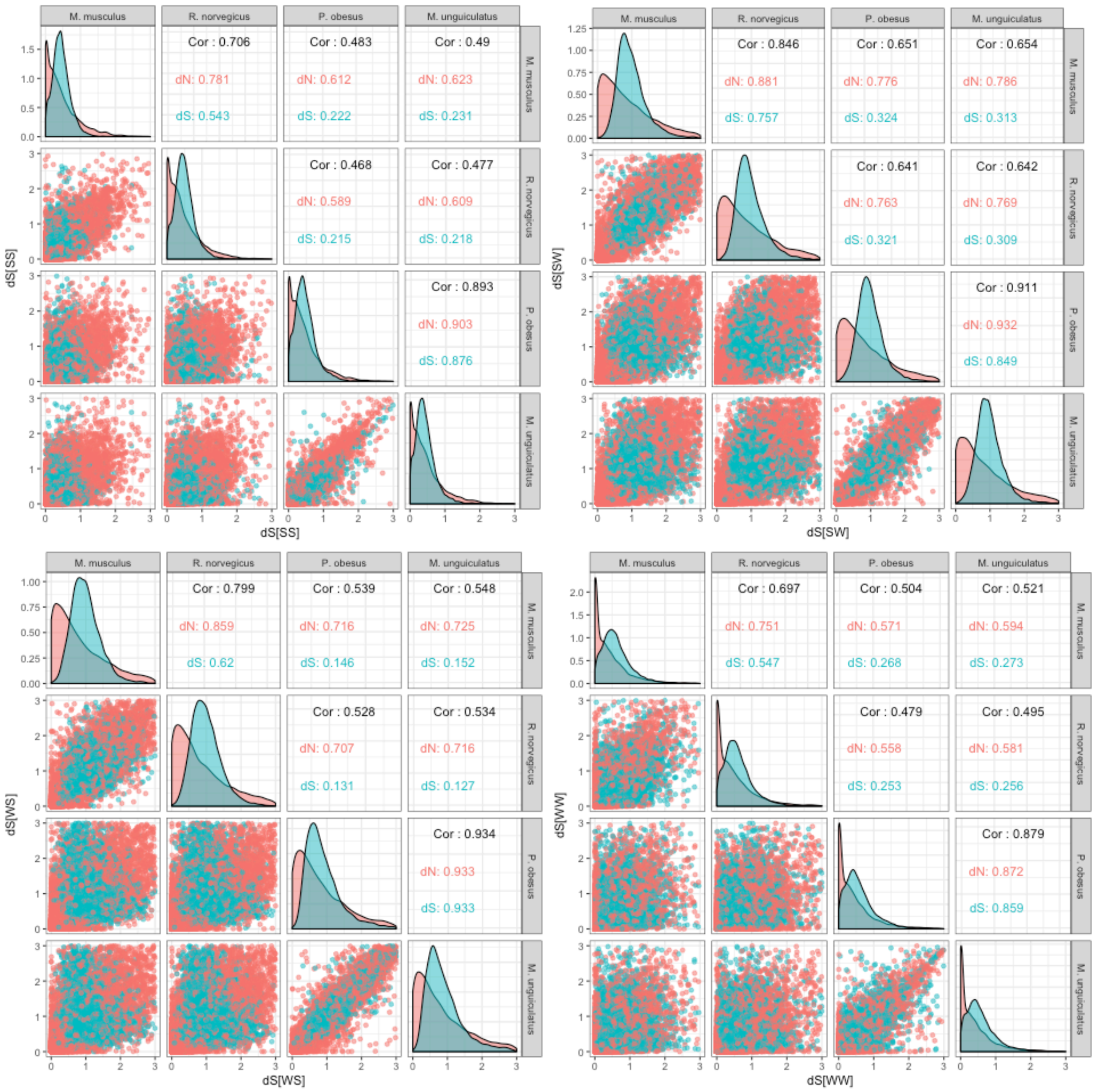

Supplementary Figure 16

Gene-by-gene pairwise correlation in the normalised substitution rate between each of the four species for each of the four mutational categories (SS, SW, WS, WW). The synonymous substitution rate (dS) is shown in teal, the nonsynonymous substitution rate (dN) in red. The plots in upper diagonal show the Spearman's rank correlation coefficient for each of the comparisons. Note the low correlations (~0.1) in dS for the WS substitution rates between the gerbil and the murid species.

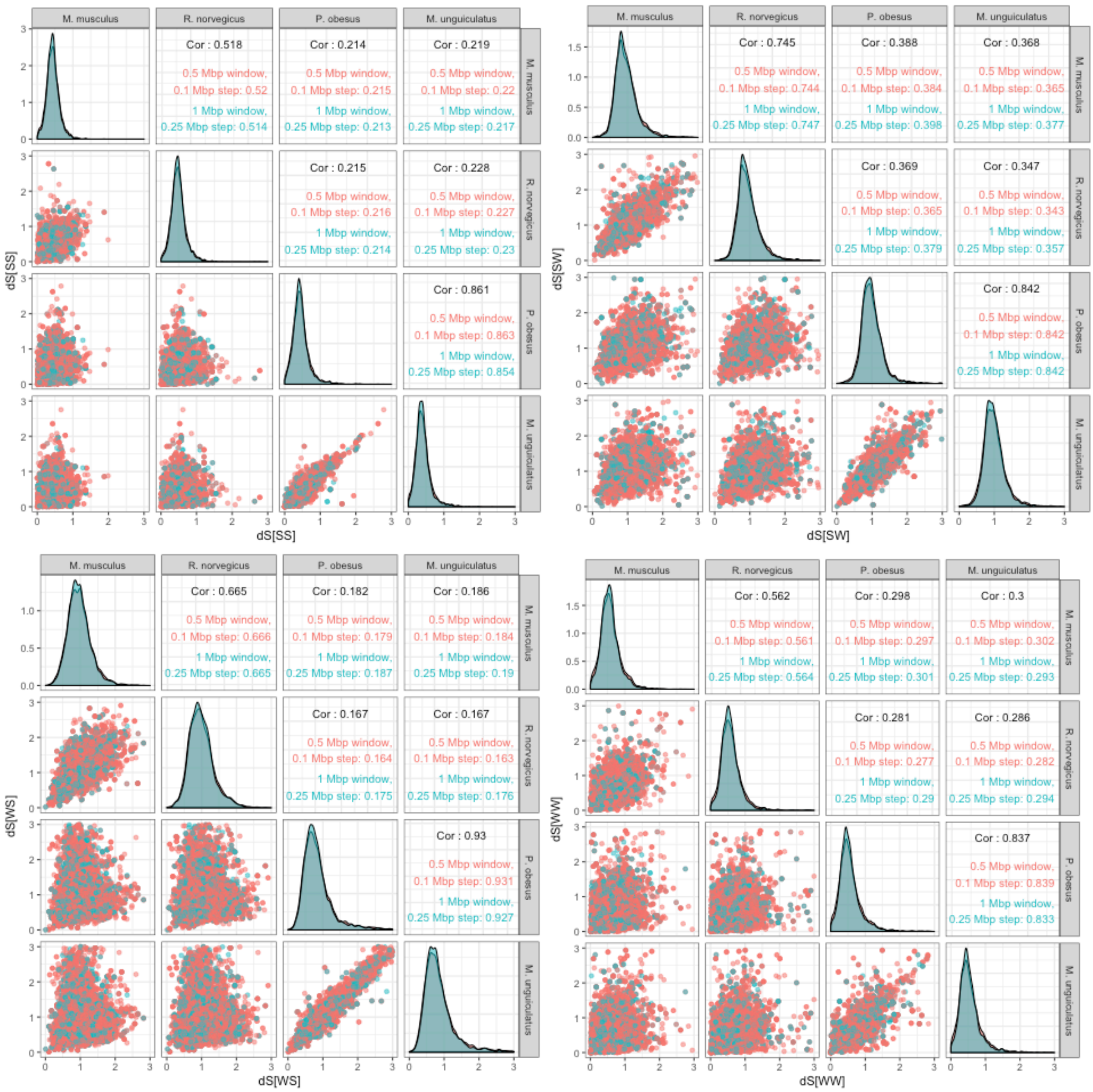

Supplementary Figure 17

Window-by-window pairwise correlation in the mean normalised synonymous substitution rate (dS) between each of the four species for two window sizes. The synonymous substitution rate (dS) is shown in teal, the nonsynonymous substitution rate (dN) in red. The plots in upper diagonal show the Spearman's rank correlation coefficient for each of the comparisons. Note the low correlations (~0.2) in dS for the WS substitution rates between the gerbil and the murid species.

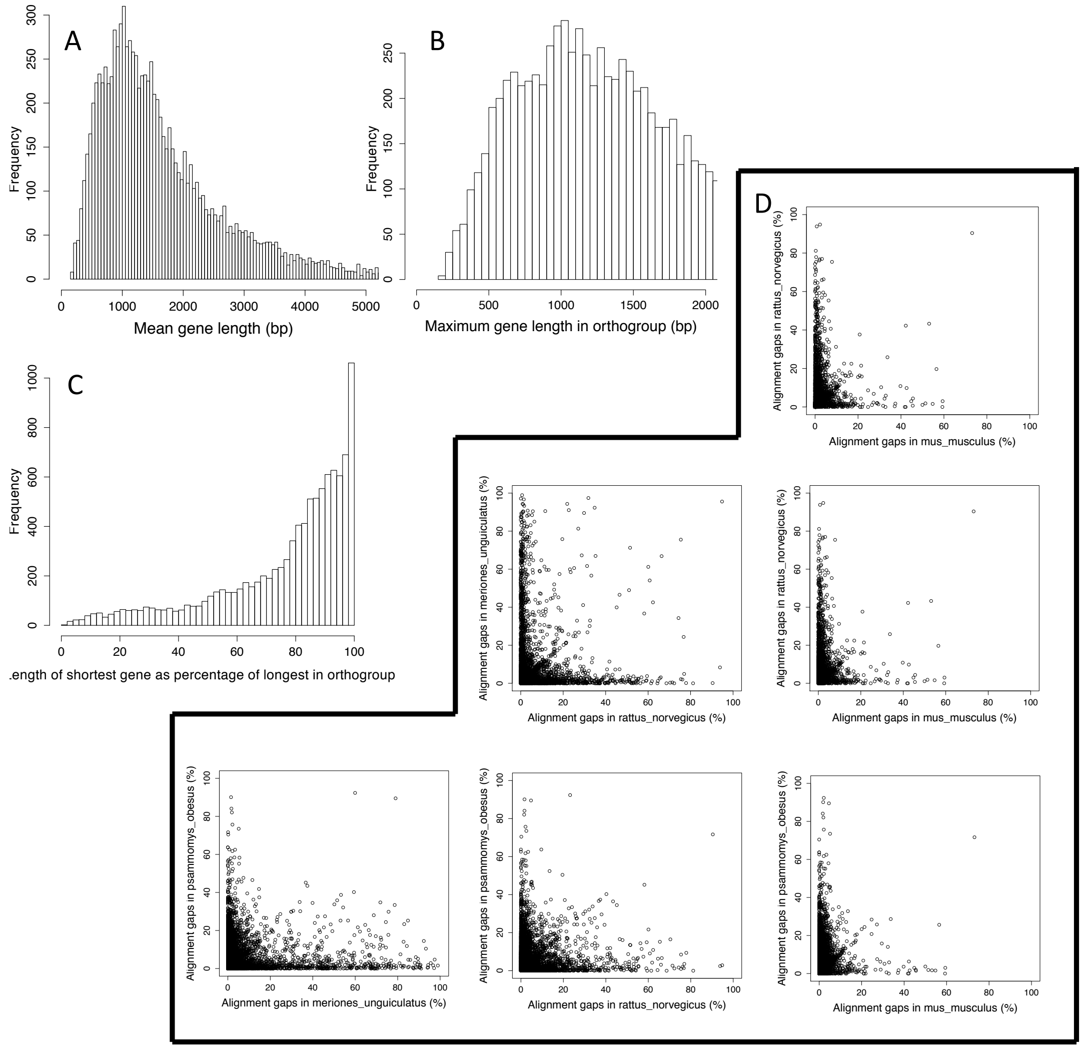

Supplementary Figure 18.

Quality of the multiple sequence alignments of the orthogroups after cleaning with HmmCleaner. (A) Distribution of mean size of the sequences in each orthogroup after the removal of gaps, limited at 5000 bp. (B) Size of the longest sequence in each orthogroup after removal of gaps, limited at 2000 bp. (C) Size of the shortest sequence in each orthogroup as percentage of the longest sequence after removal of gaps. (D) Pairwise comparison of the number of gaps in each alignment between species; the number of gaps is represented as a percentage of longest gene in the orthogroup after removal of gaps; only *Mus musculus*, *Rattus norvegicus*, *Psammomys obesus* and *Meriones unguiculatus* are shown.

Supplementary Tables

Supplementary Table 1

Number and percentage of 8,809 genes with an outlying dS for each mutational category, defined as being larger than 2.5 times the average for the respective species and mutational category.

species	WS	SW	SS	WW
Psammomys obesus	360<br>4.09%	63<br>0.72%	25<br>0.28%	100<br>1.14%
Meriones unguiculatus	387<br>4.39%	78<br>0.89%	20<br>0.23%	97<br>1.10%
Mus musculus	64<br>0.73%	101<br>1.15%	5<br>0.06%	67<br>0.76%
Rattus norvegicus	65<br>0.74%	92<br>1.04%	11<br>0.12%	66<br>0.75%

Supplementary Table 2

Normalised dS for three mutational categories (SW, WW and SS) for genes classed as outlier in dS_{WS} and for those that are not. Test statistics (W and P-value) refer to one-sided Wilcoxon rank-sum tests with continuity correction between the two groups, testing whether dS values are greater in outlier genes than in the non-outlier genes.

Species	dS Category	Median for dS_{WS} outliers	Median for other genes	W	P-value
M. unguiculatus	SW	1.08	0.94	1927853.5	5.44E-10
M. unguiculatus	WW	1.20	0.47	2704195	3.01E-107
M. unguiculatus	SS	1.04	0.38	3027680	6.05E-180
M. musculus	SW	1.11	0.91	370136	4.20E-06
M. musculus	WW	1.16	0.54	453630	5.02E-18
M. musculus	SS	0.85	0.43	495088	1.22E-26
P. obesus	SW	1.08	0.94	1810883.5	4.17E-10
P. obesus	WW	1.27	0.49	2559071	2.72E-107
P. obesus	SS	1.19	0.42	2840611	5.95E-172
R. norvegicus	SW	1.43	0.91	435511	6.40E-14
R. norvegicus	WW	1.37	0.54	483011	1.08E-22
R. norvegicus	SS	1.14	0.47	506411	7.25E-28

Supplementary Table 3

Normalised dN for four mutational categories (WS, SW, WW and SS) between genes classed as outlier in dS_{WS} and those that are not. Test statistics (W and P-value) refer to one-sided Wilcoxon rank-sum tests with continuity correction between the two groups, testing whether the dN values are greater in outlier genes than in the non-outlier genes.

Species	dN Category	Median in dS_{WS} outliers	Median for other genes	W	P-value
P. obesus	WS	1.59	0.65	2182993	6.52E-45
P. obesus	SW	0.58	0.71	1376346	1.00
P. obesus	WW	0.36	0.26	1686106	2.35E-04
P. obesus	SS	0.58	0.31	2033286	1.06E-27
M. unguiculatus	WS	1.54	0.64	2289274	9.66E-42
M. unguiculatus	SW	0.56	0.69	1471405	1.00
M. unguiculatus	WW	0.27	0.22	1767952	2.35E-03
M. unguiculatus	SS	0.62	0.30	2155102	3.25E-27
M. musculus	WS	0.91	0.67	317627	0.03
M. musculus	SW	0.67	0.72	262998	0.80
M. musculus	WW	0.22	0.24	272799	0.64
M. musculus	SS	0.26	0.31	259840	0.84
R. norvegicus	WS	1.69	0.66	394010	3.79E-08
R. norvegicus	SW	1.14	0.73	368550	1.81E-05
R. norvegicus	WW	0.63	0.29	359798	1.07E-04
R. norvegicus	SS	0.80	0.32	365114	3.72E-05

Supplementary Table 4

Distance between of dS_{WS} outliers in each species. For each species, we compared the observed distance measurements to a null distribution inferred from randomising which genes are outliers in permutations (1,000,000 permutations per species, the number of outliers per species is given in Supplementary Table 1). The P-values are calculated as the proportion of permutations where the permutation measurement is more extreme than the observation measurement. "Islands" refer to runs of more than one neighbouring outlier genes

Species	*Measurement*	*Observed*	*Mean of permutations*	*P-value*
P. obesus	Mean distance between outliers (Mbp)	5.39	6.43	1.00E-06
P. obesus	Mean number of intervening genes between outliers	20.84	23.10	5.00E-06
P. obesus	Number of islands	48.00	14.05	0
P. obesus	Average number of genes in island	3.19	2.04	0
P. obesus	Maximum distance between outliers, averaged across chromosomes (Mbp)	41.26	29.13	1.00E-06
P. obesus	Maximum number of intervening genes between outliers, across chromosomes	142.16	78.43	0
M. unguiculatus	Mean distance between outliers (Mbp)	5.14	6.02	4.00E-06
M. unguiculatus	Mean number of intervening genes between outliers	19.89	21.58	2.40E-05
M. unguiculatus	Number of islands	50.00	16.18	0
M. unguiculatus	Average number of genes in island	3.28	2.05	0
M. unguiculatus	Maximum distance between outliers, averaged across chromosomes (Mbp)	43.22	28.19	0
M. unguiculatus	Maximum number of intervening genes between outliers, across chromosomes	141.11	74.74	0
M. musculus	Mean distance between outliers (Mbp)	17.61	23.57	0.015
M. musculus	Mean number of intervening genes between outliers	62.05	91.79	0.001
M. musculus	Number of islands	2.00	0.45	0.010
M. musculus	Average number of genes in island	2.00	2.01	0.009
M. musculus	Maximum distance between outliers, averaged across chromosomes (Mbp)	39.97	44.92	0.783

M. musculus	Maximum number of intervening genes between outliers, across chromosomes	135.14	163.01	0.908
R. norvegicus	Mean distance between outliers (Mbp)	22.34	23.38	0.361
R. norvegicus	Mean number of intervening genes between outliers	89.02	90.96	0.422
R. norvegicus	Number of islands	0.00	0.47	0.378
R. norvegicus	Average number of genes in island	NA	2.01	NA
R. norvegicus	Maximum distance between outliers, averaged across chromosomes (Mbp)	53.60	44.86	0.082
R. norvegicus	Maximum number of intervening genes between outliers, across chromosomes	213.64	162.55	0.010

Supplementary Table 5

Number of "regions" of high dS_{WS}, defined as groups of one or more overlapping windows where the median dS_{WS} was greater than 2.5 the average dS_{WS} for each of the species, respectively, and the number of the dS_{WS} outlier genes represented in the regions.

Species	Window size (Mbp)	Window Step (Mbp)	Number of Regions	Number of outlier genes represented
P. obesus	0.5	0.1	18	115 (32%)
P. obesus	1	0.25	17	123 (34%)
M. unguiculatus	0.5	0.1	21	128 (33%)
M. unguiculatus	1	0.25	17	118 (30%)
R. norvegicus	0.5	0.1	0	0
R. norvegicus	1	0.25	0	0
M. musculus	0.5	0.1	0	0
M. musculus	1	0.25	0	0

Supplementary Table 6

Number of dS_{WS} outlier genes mapping to the 5 Mbp at the start and the end of mouse chromosomes. Test statistics refer Pearson's Chi-squared tests with Yates' continuity correction.

species	outlier	non-telomeric	telomeric	X	d.f.	p
P. obesus	non-outlier	7902 (94%)	547 (6%)	94.63	1	2.29E-22
P. obesus	outlier	288 (80%)	72 (20%)			
M. unguiculatus	non-outlier	7873 (93%)	549 (7%)	74.04	1	7.65E-18
M. unguiculatus	outlier	317 (82%)	70 (18%)			
M. musculus	non-outlier	8130 (93%)	615 (7%)	1.24E-28	1	1
M. musculus	outlier	60 (94%)	4 (6%)			
R. norvegicus	non-outlier	8127 (93%)	617 (7%)	1.01	1	0.31
R. norvegicus	outlier	63 (97%)	2 (3%)			

Supplementary Table 7

Number of regions with high average dS_{WS} mapping to the 5 Mbp at the start and the end of mouse chromosomes.

species	window_category	non-telomeric	telomeric
P. obesus	0.5 Mbp window, 0.1 Mbp step	10 (56%)	8 (44%)
P. obesus	1 Mbp window, 0.25 Mbp step	8 (47%)	9 (53%)
M. unguiculatus	0.5 Mbp window, 0.1 Mbp step	14 (67%)	7 (33%)
M. unguiculatus	1 Mbp window, 0.25 Mbp step	9 (53%)	8 (47%)

Supplementary Table 8

GC3 ranking of dS_{WS} outlier and non-outlier genes in the HOGs they were assigned to.

Species	Rank	dS_{WS} outliers		Other genes		Total
			(%)		(%)	
P. obesus	Rank <= 3	90	49.2	93	50.8	183
M. unguiculatus	Rank <= 3	108	51.9	100	48.1	208
M. musculus	Rank <= 3	6	10.3	52	89.7	58
R. norvegicus	Rank <= 3	2	3.1	63	96.9	65
P. obesus	Rank > 3	203	3.1	6349	96.9	6552
M. unguiculatus	Rank > 3	199	3	6328	97	6527
M. musculus	Rank > 3	44	0.7	6633	99.3	6677
R. norvegicus	Rank > 3	57	0.9	6613	99.1	6670
P. obesus	Rank = 1	38	70.4	16	29.6	54
M. unguiculatus	Rank = 1	47	71.2	19	28.8	66
M. musculus	Rank = 1	3	21.4	11	78.6	14
R. norvegicus	Rank = 1	1	10	9	90	10
P. obesus	Rank > 1	255	3.8	6426	96.2	6681
M. unguiculatus	Rank > 1	260	3.9	6409	96.1	6669
M. musculus	Rank > 1	47	0.7	6674	99.3	6721
R. norvegicus	Rank > 1	58	0.9	6667	99.1	6725

Supplementary Table 9

Number of genes in the previously known high-GC region with a synonymous substitution rate higher than the 95th and 99th percentile value in the set of 8,909 orthologous genes representing the genome-wide distribution.

Species	Category	Quantile	Number of genes	Quantile value	Number of genes in quantile
P. obesus	SS	0.95	27	0.3	27 (100%)
P. obesus	SW	0.95	27	0.21	27 (100%)
P. obesus	WS	0.95	27	0.44	27 (100%)
P. obesus	WW	0.95	27	0.34	27 (100%)
M. unguiculatus	SS	0.95	24	0.3	24 (100%)
M. unguiculatus	SW	0.95	24	0.2	24 (100%)
M. unguiculatus	WS	0.95	24	0.47	24 (100%)
M. unguiculatus	WW	0.95	24	0.34	24 (100%)
P. obesus	SS	0.99	27	0.45	27 (100%)
P. obesus	SW	0.99	27	0.28	27 (100%)
P. obesus	WS	0.99	27	0.9	27 (100%)
P. obesus	WW	0.99	27	0.59	27 (100%)
M. unguiculatus	SS	0.99	24	0.45	24 (100%)
M. unguiculatus	SW	0.99	24	0.28	24 (100%)
M. unguiculatus	WS	0.99	24	0.92	24 (100%)
M. unguiculatus	WW	0.99	24	0.59	23 (96%)

Supplementary Table 10

Accession ID of predicted genome annotations used in analysis.

Species name	Common name	genome-build	genome-date	genome-build-accession	genebuild-last-updated
Cavia porcellus	Domestic guinea pig	Cavpor3.0	2008-03	NCBI:GCA_000151735.1	2017-07
Chinchilla lanigera	Long-tailed chinchilla	ChiLan1.0	2012-05	NCBI:GCA_000276665.1	2017-07
Dipodomys ordii	Ord's kangaroo rat	Dord_2.0	2014-12	NCBI:GCA_000151885.2	2017-07
Fukomys damarensis	Damara mole rat	DMR_v1.0	2014-09	NCBI:GCA_000743615.1	2017-02
Homo sapiens	Human	GRCh38.p12	2013-12	NCBI:GCA_000001405.27	2018-07
Ictidomys tridecemlineatus	Thirteen-lined ground squirrel	SpeTri2.0	2011-11	NCBI:GCA_000236235.1	2017-07
Jaculus jaculus	Lesser Egyptian jerboa	JacJac1.0	2012-07	NCBI:GCA_000280705.1	2017-02
Meriones unguiculatus	Mongolian gerbil	MunDraft-v1.0	2017-06	NCBI:GCA_002204375.1	2018-12
Mus musculus	Mouse	GRCm38.p6	2012-01	NCBI:GCA_000001635.8	2018-09
Nannospalax galili	Upper Galilee mountains blind mole rat	S.galili_v1.0	2014-05	NCBI:GCA_000622305.1	2017-02
Octodon degus	Degu	OctDeg1.0	2012-05	NCBI:GCA_000260255.1	2017-07
Rattus norvegicus	Rat	Rnor_6.0	2014-07	NCBI:GCA_000001895.4	2017-01
